# Prevalence of Behavior Changing Strategies in Fitness Video Games: Theory-Based Content Analysis

**DOI:** 10.2196/jmir.2403

**Published:** 2013-05-07

**Authors:** Elizabeth Jane Lyons, Claire Hatkevich

**Affiliations:** ^1^Institute for Translational SciencesSchool of MedicineUniversity of Texas Medical BranchGalveston, TXUnited States; ^2^Department of PsychologyUniversity of DelawareNewark, DEUnited States

**Keywords:** video game, theory, content analysis, fitness, physical activity, exergame

## Abstract

**Background:**

Fitness video games are popular, but little is known about their content. Because many contain interactive tools that mimic behavioral strategies from weight loss intervention programs, it is possible that differences in content could affect player physical activity and/or weight outcomes. There is a need for a better understanding of what behavioral strategies are currently available in fitness games and how they are implemented.

**Objective:**

The purpose of this study was to investigate the prevalence of evidence-based behavioral strategies across fitness video games available for home use. Games available for consoles that used camera-based controllers were also contrasted with games available for a console that used handheld motion controllers.

**Methods:**

Fitness games (N=18) available for three home consoles were systematically identified and play-tested by 2 trained coders for at least 3 hours each. In cases of multiple games from one series, only the most recently released game was included. The Sony PlayStation 3 and Microsoft Xbox360 were the two camera-based consoles, and the Nintendo Wii was the handheld motion controller console. A coding list based on a taxonomy of behavioral strategies was used to begin coding. Codes were refined in an iterative process based on data found during play-testing.

**Results:**

The most prevalent behavioral strategies were modeling (17/18), specific performance feedback (17/18), reinforcement (16/18), caloric expenditure feedback (15/18), and guided practice (15/18). All games included some kind of feedback on performance accuracy, exercise frequency, and/or fitness progress. Action planning (scheduling future workouts) was the least prevalent of the included strategies (4/18). Twelve games included some kind of social integration, with nine of them providing options for real-time multiplayer sessions. Only two games did not feature any kind of reward. Games for the camera-based consoles (mean 12.89, SD 2.71) included a greater number of strategies than those for the handheld motion controller console (mean 10.00, SD 2.74, *P*=.04).

**Conclusions:**

Behavioral strategies for increasing self-efficacy and self-regulation are common in home console fitness video games. Social support and reinforcement occurred in approximately half of the studied games. Strategy prevalence varies by console type, partially due to greater feedback afforded by camera-based controllers. Experimental studies are required to test the effects of these strategies when delivered as interactive tools, as this medium may represent an innovative platform for disseminating evidence-based behavioral weight loss intervention components.

## Introduction

Insufficient physical activity and poor dietary habits contribute to obesity [1-3], which in turn contributes to numerous negative health outcomes [4,5]. The current and persistent high prevalence of obesity in the United States [6] will require innovative solutions to this seemingly intractable problem.

A potential platform for those solutions is the video game console. Due to their popularity and potential for influencing health, fitness-themed video games have recently attracted substantial research attention [7-13]. Fitness games are a subset of motion-controlled or “active” games that specifically emphasize fitness/exercise content, extending the traditional format of workout videos by increasing their interactivity. For example, the games may allow players to adjust their difficulty level and choose preferred exercises. Because the games use motion-sensing controllers, they also allow for feedback on user performance. Studies of fitness games have found that they are capable of producing a range of energy expenditure values, from light to vigorous intensity physical activity [14-19]. Though these games mostly are targeted towards and appeal to middle-aged women [20], they appear to be acceptable across a range of ages and in both genders [11,13,17,21].

Little is known about their content and how differences in content may affect player behavior. Specifically, many fitness games include interactive behavioral tools. These tools typically use strategies that are hallmarks of successful weight loss interventions such as modeling and goal-setting [22-26]. There is evidence that increased use of behavioral strategies can improve the outcomes of weight loss trials [27,28], but programs that use these strategies are quite costly [29]. Using interactive technological tools to deliver behavioral weight loss interventions has been shown to be less costly and as or more efficacious [30]. Video-game based tools may offer an opportunity to deliver portions of clinical weight loss programs in settings previously unable to receive them due to cost.

### Behavioral Strategies to Enhance Self-Efficacy

Standard behavioral weight loss interventions typically include a large number of strategies aimed at behavior change. Recent meta-analyses and meta-regressions have identified several strategies that appear to be particularly effective for promoting weight loss and physical activity [25,31]. Many of these strategies target increases in self-efficacy, or confidence in one’s ability to perform a specific course of action, which is strongly related to beneficial weight loss intervention effects.

A strategy from face-to-face interventions used commonly in video games is modeling, also called observational learning or vicarious experience. In this strategy, an individual is observed while performing targeted activities; the player learns how to perform the activities by watching the model. Models can be computer-generated agents such as virtual trainers, or they can be representations of the player him/herself in video or computer-generated avatar form. Virtual self-modeling using video or avatars may be particularly powerful. Several studies have found that watching a virtual self eat or exercise (as compared to watching a virtual other) can affect later real-world eating and exercise [32,33].

Another very common strategy with proven effectiveness is provision of feedback on performance [25]. One of the benefits of technology-based intervention is that feedback on performance and progress can be provided automatically and immediately. Both general (calorie burn, number of sessions completed) and specific (accuracy of individual exercises) feedback may be provided by a fitness video game. Some camera-based games also provide players with visual feedback in the form of real-time video footage of their movements.

Both modeling and feedback can be integrated with guided practice, also known as guided mastery. Guided practice provides a chance for an individual to practice enacting a skill while receiving instruction. In fitness video games, guided practice may consist of players simultaneously receiving instruction, watching a model perform a set of exercises, enacting the movements themselves, and receiving feedback on the accuracy of their practice movements. Verbal persuasion as to the player’s competence (ie, encouragement) may also be integrated with these strategies. Verbal persuasion can occur during exercise (“You can do it!”) or afterward as part of evaluative feedback (“Good job, you really have a knack for this.”).

### Behavioral Strategies to Enhance Self-Regulation

Self-regulation is often used synonymously with self-control and encompasses self-corrective adjustments towards some purpose [34]. Self-regulation strategies are also central to behavior change interventions. In fact, self-regulation strategies such as self-monitoring of caloric intake, physical activity, and weight are among the strongest predictors of weight loss [22]. Strategies that influence self-regulation may include self-monitoring, goal-setting, action planning, and self/social comparison.

Many current fitness video games track physical activity frequency, duration, and caloric expenditure. Some also track weight (via use of a balance board controller) and fitness (via heart rate and/or number of repetitions during periodic fitness tests). This information is typically provided as graphs, progress bars, and leaderboards. Players may also earn achievements or trophies (ie, virtual badges) for reaching caloric expenditure or frequency cut-points. These tracking systems are more sophisticated than physical activity self-monitoring in typical weight loss interventions, which is often self-reported. Automated measurement and feedback from monitoring systems similar to these have been found to be very effective in weight loss trials [35-37].

Goal-setting provides a framework for self-monitoring and feedback, which are specific to a particular goal (eg, eating 1500 calories per day). Goals can also influence performance in multiple ways, including via motivating greater effort and by directing effort towards task-relevant activities [38]. Goals can be integrated with other strategies to enhance their effectiveness; for example, successful attainment of subgoals could be reinforced by a reward system. Action planning to reach the goals may include creating weekly schedules for play sessions, choosing from suggested content for each session, and following preset guidelines (eg, a 30-day challenge). Diagnostic pretests can be used in interactive media to determine an individual’s current level of fitness, set proximal and distal goals, and set a time frame for accomplishing those goals. These pretests collect information for use in tailoring later content much like baseline assessments and initial individual sessions would in a face-to-face intervention.

Comparison is also an important component of feedback that affects self-monitoring. By viewing workout calendars and graphs of progress, individuals can compare their current performance to past performance. Inclusion of multiplayer graphs and leaderboards allow individuals to compare their performance to that of other individuals. Monitoring one’s progress and comparing it to benchmarks allows individuals to quickly adapt their behavior (eg, exercising more on a day in which calorie intake goals were not met). Comparison is also very effective in increasing self-efficacy for exercise [25].

### Other Social-Cognitive Behavioral Strategies: Reinforcement and Social Integration

Several additional behavioral strategies used in weight loss studies may be implemented in fitness video games, such as reinforcement and social integration. Reinforcement is a key feature of video games that is also used in fitness video games. These games may include in-game and/or console-based rewards. In-game rewards typically are virtual badges of some kind that are achieved by reaching preset goals. These badges may also be tied to unlocking new parts of the game, such as new environments, music, or exercises. Console-based rewards are also virtual badges, but these are tied to an individual’s online account and are visible to others on online game services. In addition to serving as rewards and indicators of progress, these virtual badges (called achievements on Xbox Live and trophies on PlayStation Network) also indicate group affiliations and individual status by displaying to others what games individuals play and how they play them. It has been hypothesized that virtual badges may be more motivating when they serve these additional social purposes [39,40], and thus console-based rewards should be considered separately from in-game awards.

Other methods of integrating social support and influence may also affect physical activity and weight loss behaviors. Social support from family, friends, and other participants is often a feature of in-person and Internet-mediated programming. Social play is also one of the major uses and gratifications of video game play [41]. Generalized social integration may include leaderboards or asynchronous multiplayer play (eg, trying to beat a previous high score). Synchronous multiplayer features allow players to cooperatively or competitively play with one another at the same time either in-person or over the Internet. Because feelings of relatedness to others are central to intrinsic motivation (the desire to engage in a behavior for its own sake in the absence of external pressures/rewards) [42], more social play options may produce greater adherence to use of the game.

### Behavioral Strategies and Active Video Games

Though we are unaware of any studies of behavioral strategies in commercially available video games, these strategies have been used in technology-enhanced interventions using activity monitors, PDAs, and mobile phones. Some activity monitoring systems provide detailed graphs and leaderboards to show progress over time and in comparison to others (eg, *BodyMedia*, *FitBit*). Several studies have found that technology-enhanced self-monitoring produces greater adherence and thus greater intervention effects when compared to traditional paper self-monitoring [43-45]. Thus, more than simply encouraging greater energy expenditure during screen time, fitness video games hold the potential for intervening on intermediate psychosocial variables that affect weight. There is a need for a better understanding of the prevalence of game tools incorporating these strategies.

The purpose of this study was to characterize currently available fitness games based on the prevalence of interactive tools using behavioral strategies from successful weight loss interventions. A secondary goal was to compare behavioral tools across two different types of consoles—those that use camera-based controllers and those that use handheld controllers. We hypothesized that games for the camera-based consoles would include more behavioral strategies than those available for a handheld controller-based console. Camera-based controls may allow for the use of more types of strategies because they can measure player movement more precisely and provide video feedback.

## Methods

### Console and Controller Descriptions

The three major home consoles for video game play (as of August, 2012) are the Microsoft Xbox 360, Nintendo Wii, and Sony PlayStation 3. Each console uses a different method of evaluating player movement during play of fitness video games.

The Xbox 360 uses its Kinect peripheral, which is a camera that analyzes body movement with no need for handheld controllers. Because the Kinect is camera-based, it allows for still pictures and videos to be used by game software. Real-time video of player movement can be shown on the screen and integrated into game scenes.

The Wii uses two handheld controllers (Wiimote and Nunchuk) and a balance board to evaluate player movement. Different games use different combinations and configurations of these three controllers to measure arm, leg, and body movement. The balance board can detect shifts in weight, allowing it to be used for weigh-ins as well.

The PlayStation 3 uses the PlayStation Eye and Move peripheral devices. The Eye is a camera, and the Move controllers are similar to Wiimotes. Thus, this system is a hybrid of the two mentioned above. Still pictures and videos can appear on screen, and the player also holds a tangible controller.

### Game Inclusion

A systematic search was conducted to find available fitness games for home consoles. Two of the largest sellers of video games, Amazon.com and Gamestop.com, were searched using four search terms: “fit,” “fitness,” “active,” and “workout” (for Amazon, these searches were conducted specifically within the video game category). After eliminating duplicate entries, a total of 40 unique titles were found. To be included in the analysis, games must have been the most recent game available within a franchise (eg, *Jillian Michaels Fitness Adventure* is the 2012 entry into the Jillian Michaels Fitness franchise), and they must have primarily a fitness focus rather than a party game or minigame collection focus (excluded: games in the Active Life franchise, *Boot Camp*, *Family Party: Fitness Fun*, *Kid Fit Island Resort*, *Nickelodeon Fit*). After excluding multiple games in a franchise, 23 games remained; six were eliminated due to a primary focus as a party/minigame game. To this total of 17 games, an additional game was added. It was deemed that a downloadable fitness pack created sufficient fitness content that *The Fight: Lights Out* could be included. Thus, the final total of games was 18. The preliminary search was conducted in November 2011. A follow-up search was conducted in June 2012 to update franchises to their latest iterations (*Jillian Michaels Fitness Adventure* and *Zumba Fitness Rush* replaced earlier versions of both games). We chose to include *My Fitness Coach* despite the existence of *My Fitness Coach 2* because it was an updated re-release of the Xbox/PC game *Yourself Fitness*, whereas the US release of *My Fitness Coach 2* was a re-branded version of the *New U* series of games.

In cases in which a game was ported to multiple consoles, each version of the game was play-tested. The version that included the highest number of behavioral tools was included in this analysis (for *EA Sports Active 2* and *UFC Personal Trainer*, PlayStation 3 and Wii versions were excluded and the Xbox360 version used; for *Fit in Six* and *Get Fit With Mel B*, Wii versions were excluded and the PlayStation 3 version was used). These games were typically identical across consoles except for slight changes made possible by different controller systems, which allowed inclusion of several behavioral tools (ie, viewing real-time video of the self on the screen).

Included games played on an Xbox 360 console were: *The Biggest Loser: Ultimate Workout*, *EA Sports Active 2*, *Jillian Michaels Fitness Adventure*, *UFC Personal Trainer*, *Your Shape: Fitness Evolved 2012*, and *Zumba Fitness Rush*. Games played on a PlayStation 3 console were: *Fix in Six*, *Get Fit With Mel B*, and *The Fight: Lights Out* (with downloaded Fitness Pack content). Games played on a Nintendo Wii console were: *10 Minute Solution*, *Daisy Fuentes Pilates*, *Exerbeat*, *Gold’s Gym Cardio Workout*, *Gold’s Gym Dance Workout*, *My Fitness Coach*, *New U Fitness First Yoga and Pilates*, *Walk It Out!*, and *Wii Fit Plus*.

### Coding List and Procedure

Coding procedures for this study followed both theory-based and grounded theory strategies. The 2 coders began with a coding list based on Abraham and Michie’s taxonomy of behavior change strategies [46] as well as strategies used to increase self-efficacy from Bandura and from the research literature [23,25,31]. Then, using grounded theory, codes were added and adapted throughout the playtesting process to reflect the content of the games [47]. This process was similar to that used in our previous content analyses [48].

**Figure 1:**
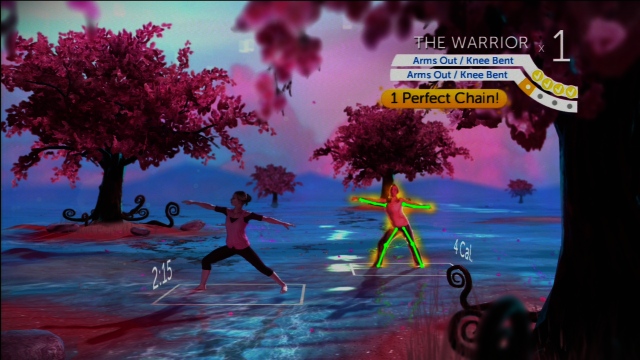
An example of self-efficacy behavioral strategies in Your Shape: Fitness Evolved 2012.

**Figure 2:**
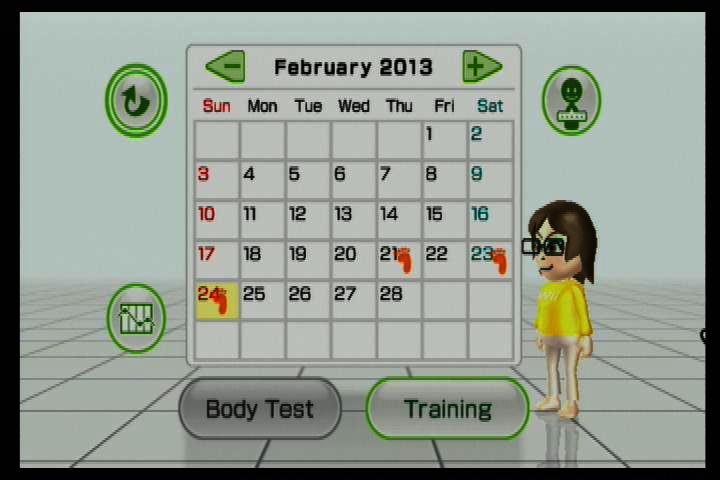
An example of a workout calendar from Wii Fit Plus.

A final list of 17 codes is displayed in Table 1. A screenshot from *Your Shape: Fitness Evolved 2012* in Figure 1 shows an example of modeling by a virtual trainer, virtual self-modeling (showing the individual performing the exercise), accuracy feedback (checkmarks in the top corner for each portion of the move performed correctly, green lines superimposed on the body to show how the limbs are bent/straight, glowing body to indicate a “combo” of correct movements in a row), and video of body movement. The body on the left in the screenshot is the virtual trainer, and the body on the right is a real-time video feed of the player. A screenshot from Wii Fit Plus in Figure 2 shows an example of a workout calendar. Additional descriptions of games and screenshots are available in Multimedia Appendix 1.

Each game was play-tested for at least 3 hours by each independent coder. Interrater agreement between the 2 coders was high (0.90). Cohen’s kappa was also calculated to compare coder agreement to chance (κ=.80). The 2 coders met to discuss all disagreements and decide upon appropriate final codes.

Simple descriptive statistics were calculated to investigate the prevalence of strategies in the games. To compare consoles, Student’s *t* tests were used.

**Table 1:** Code list with descriptions.

	Code	Description
**Self-efficacy**		
	Modeling by trainer	A virtual trainer performs the exercises to show proper form.
	Virtual self-modeling	An avatar or video of the player’s face and body is shown, modeling the exercise behavior.
	Guided practice	A practice or tutorial session led by a trainer is included.
	Verbal persuasion	The virtual trainer verbally encourages greater exercise self-efficacy. (“You can do it!”)
	Accuracy feedback	Specific feedback on the accuracy of individual exercises is provided.
	Performance feedback	Overall feedback on player performance during a workout session is provided.
	Calorie burn feedback	Numeric totals for estimated caloric burn are provided.
	Video of body movement	Real-time video is shown on screen (player representation may be a colored shape).
**Self-regulation**		
	Goal-setting	Players are prompted to set goals for themselves.
	Diagnostic pretest	A pretest is given to help in the selection of appropriate goals, programs, and difficulty.
	Scheduling	Players schedule workout days in advance, either manually or as part of a pre-made program.
	Workout calendar	A calendar is provided that shows a history of game play and, in some cases, upcoming scheduled workouts.
	Comparison to past	Player progress over time is shown in chart form.
**Other**		
	Social integration	Other players are incorporated into the game in some fashion (eg, leaderboards, asynchronous multiplayer).
	Multiplayer	Individuals can play together with another person at the same time either in person or via the Internet.
	In-game rewards	The game provides virtual rewards for progress.
	Console-based rewards	The game provides achievements or trophies that are shown on the console’s online service to other users.

## Results

Behavioral strategies found in each game are shown in Multimedia Appendix 2 (camera-based games) and Multimedia Appendix 3 (controller-based games). Feedback comparing current to past behavior (either performance, attendance, or estimated calorie burn) was present in all of the studied games. Modeling (17/18 games), immediate performance feedback (17/18), caloric expenditure feedback (15/18), guided practice (15/18), and a workout calendar (14/18) were included in a majority of the games. All but two of the games (16/18) included some kind of reward system, either directly in the game itself or integrated into a console-wide online virtual badge system. Few games included a pretest (5/18) or the ability to schedule future workouts (4/18).

The number of strategies used in a game were higher in the camera-based games (mean 12.89, SD 2.71) than in controller-based games (mean 10.00, SD 2.74; *P*=.04).

## Discussion

### Principal Findings

This content analysis demonstrated that interactive behavioral tools for promoting physical activity and weight loss are common in fitness video games for home consoles. Feedback, modeling, rewards, practice, self-monitoring, and reinforcement were the most prevalent strategies. Games that used camera-based controllers (Xbox 360, PlayStation 3) contained more strategies than games that used non–camera-based controllers (Wii).

Reviews and secondary analyses of behavioral weight loss trials suggest that inclusion of self-monitoring [22,26], goal-setting [22,28], action planning [23], reinforcement [23], practice [26,49], and social support and comparison [23,26,49] likely contributes to intervention success. Many fitness video games include behavioral tools that closely mimic the implementation of these behavioral strategies in face-to-face and eHealth settings. Numerous studies have tested fitness video games, but we are unaware of any that have specifically investigated possible effects of their behavioral content. Because these interactive tools closely resemble behavioral strategies used successfully in clinical weight loss interventions, games containing them may hold potential as inexpensive, highly disseminable intervention media.

The public health impact of an intervention can be conceptualized by the RE-AIM framework (Reach, Effectiveness, Adoption, Implementation, Maintenance) [50]. Increasing the reach of clinical weight loss interventions, even if the effectiveness is slightly lowered, could greatly increase their ultimate public health impact. Internet-based studies have successfully translated behavioral weight loss interventions into a more disseminable format while retaining clinically meaningful effectiveness [51]. Additional studies using electronic monitors have shown that technology-assisted self-monitoring and automatic feedback can successfully translate self-regulatory portions of these interventions to nonclinical settings [36,37]. Fitness video games could potentially be a next step in the process of further translation, as they may also be able to use interactive strategies such as virtual self-modeling to increase self-efficacy [11].

Weight loss trials that adhere to theory have been found to be more effective than those that do not [52,53]. Thus, based on theoretical conceptualizations of self-efficacy, we would expect feedback that is immediate, specific, clear, based on goals, and that includes comparisons (both to past performance and to others) to produce greater self-efficacy and physical activity [31]. Fitness video games are capable of offering richer and more extensive feedback than noninteractive exercise instruction via audio or video cues. For example, *Your Shape: Fitness Evolved 2012* superimposes lines on video of the player’s arms, trunk, and legs during yoga moves. As the player’s body aligns properly for the move, the lines change color to indicate successful performance. Non–camera-based games can also provide in-depth feedback. They typically use the placement of the handheld controller to sense whether actions are being performed precisely and provide numerical (number of repetitions) or qualitative (eg, miss/good/perfect) feedback. By immediately evaluating the precision of the player’s movements and encouraging correct form, these games may promote safe and effective home-based exercise.

Behavioral weight loss interventions often include assistive devices that help participants self-monitor progress towards their goals. These devices can include calorie books, diaries, activity monitors, scales, and online monitoring programs [38]. Many of the games studied here could be considered assistive devices, providing extensive feedback on objectively measured activity. Some upcoming games, such as *Wii Fit U*, will also be able to track lifestyle activity by incorporating pedometers.

The games studied here included a wide variety of evidence-based interactive tools. However, some effective strategies were underrepresented. Action planning, operationalized here as scheduling workouts, is associated with increased efficacy [23] but was rarely found in these games. Other, more involved forms of action planning (such as making specific action plans for different types of exercise and for overcoming barriers) were not found. Diagnostic pretests, which could serve as a method of gathering information for goal-setting, action planning, and tailoring programs to individual preferences were also rare.

Games for the camera-based Kinect and Move controllers (for the Microsoft Xbox 360 and Sony PlayStation 3) provided more tools for promoting self-efficacy, self-regulation, and weight loss than games for a console with no camera (Nintendo Wii). By its nature, a camera-based controller allows for more extensive feedback than a handheld controller that uses an accelerometer. These console differences may change with the upcoming next generation of the three major home consoles, however. The Nintendo Wii U will use a tablet-based controller that houses a camera in addition to a touch screen and motion control. Also, two of the game franchises that included the highest number of behavioral strategies, *Your Shape* and *Wii Fit*, will be available for this console. Follow-ups to the PlayStation 3 and/or Xbox 360 may have more technologically sophisticated camera controllers, which could impact the types of motions that can be interpreted by games. In addition, camera-based game apps for mobile devices have also begun to appear. For example, *Bit Breaker* for iOS uses a mobile device’s camera to sense movement and map that movement onto a simple, Pong-like game. Many mobile devices include cameras, accelerometers, and GPS, making them an attractive option for future fitness game development. For example, the smartphone game *Zombies, Run!* provides extensive performance feedback on distance and speed for overall workouts and even during specific songs the player listened to during the workout. GPS capabilities of smartphones provide more accurate and sophisticated data than are possible from current consoles. Thus, mobile games may also be a fruitful area for future research into behavioral strategies, particularly feedback.

### Limitations

This study represents a first step in identifying and measuring interactive tools in video games that may hold potential for weight loss, but its preliminary nature prevents the drawing of strong conclusions. Because this was a content analysis, we cannot determine from these data whether or not the existence of more behavioral tools in a game is associated with greater effectiveness in promoting weight loss or physical activity. The comparison across console types is also limited by our inclusion criteria, as we specifically included only the version of some games that had the most behavioral tools so as not to compare different console versions of the same game (ie, we did not include the Wii or PlayStation 3 versions of the UFC game, only the Xbox360 version).

Commercial (ie, not based in the home) exergames and mobile exergames were not included in this analysis, nor were active games that did not focus on fitness. Some motion-controlled games are now available for tablets, smartphones, and other consoles (eg, *XaviX*). Further, many nongame applications for smartphones and tablets have been “gamified” and thus, though they are not true video games, they include many game-like aspects (eg, virtual badges, leaderboards). Some nonfitness games also include some integration of fitness content, such as calorie tracking in dance simulation games. These other games and applications and their incorporation of behavioral strategies are worth research attention in the future.

### Conclusions

A broad range of fitness video games are available for home use. All of the studied games incorporated at least five behavioral strategies similar to those used in clinical weight loss interventions. It is premature to draw conclusions as to whether games with more behavioral strategies may produce greater physical activity or weight loss; the potential of specific strategies or groups of strategies for increasing the effectiveness of active video games deserves study in both cross-sectional laboratory studies and in randomized controlled trials. There is a need for careful attention to game content prior to the implementation of active games in health promotion programs. Future studies should closely investigate how behavioral tools in fitness video games are used by participants and measure intermediate process and mediating variables to better understand the possible impacts of interactive behavioral tools.

## Multimedia Appendix 1

Brief descriptions and screenshots from all of the included games.

## Multimedia Appendix 2

Behavioral strategies in camera-based games.

## Multimedia Appendix 3

Behavioral strategies in controller-based games.

## Abbreviations

iOS: “i” Operating System (operating system used by iPhones and iPads)
PDA: Personal Digital Assistant
RE-AIM: Reach Effectiveness Adoption Implementation Maintenance
UFC: Ultimate Fighting Championship
