# Supplementary material for: Prevalence of Behavior Changing Strategies in Fitness Video Games: Theory-Based Content Analysis
Source: J Med Internet Res. 2013 May 7;15(5):e81. doi: 10.2196/jmir.2403 (PMC3650924; doi:10.2196/jmir.2403)

Multimedia Appendix 1. Game descriptions and screen shots.

Screen shots were taken in February, 2012, using an Avermedia video capture device. When there is a player shown, the player is the first author. Component connections were attempted for all consoles, but difficulties with the Playstation 3 led to lower quality output than from the Xbox 360 and Wii. The Wii is not a high definition console, so graphical differences between it and the Xbox 360 are true differences. The Playstation 3 is capable of 1080 high definition like the Xbox 360, but graphical quality appears lower here due to a poorer connection to the capture device. The poor connection caused many of the written words in the pictures for Fit In Six, Get Fit With Mel B, and The Fight: Lights Out to appear blurry and pixelated. All of these three games can be played in high definition, and the writing does not appear blurred when played without the poor connection we unfortunately had to use for these pictures.

Below we present three screenshots for each of the games tested in this study with a brief explanation of what they show.

Camera-based games

The Biggest Loser: Ultimate Workout

The Biggest Loser: Ultimate Workout (released by THQ in 2010 for the Xbox 360) uses the Kinect camera-based controller to evaluate player movement. This game includes pre-scheduling of workouts towards achieving goals. “BerryzKobold” is the Xbox360 GamerTag (ID that remains constant across all games) of the player.

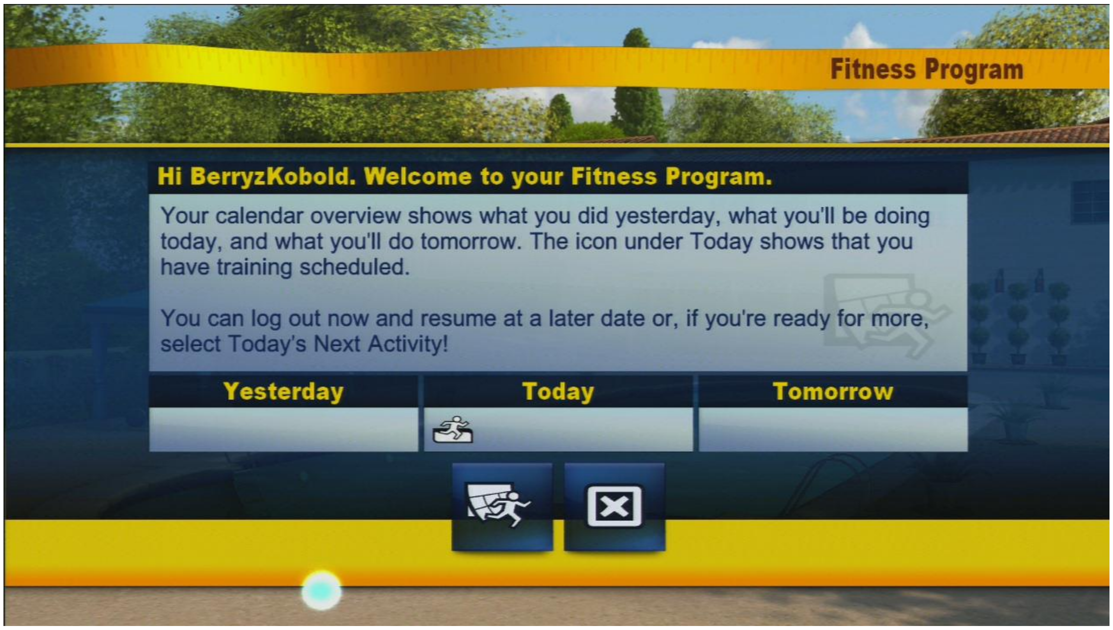

During workouts, the computer-generated virtual trainer (one of two celebrity trainers from the television show The Biggest Loser; here we show Jillian Michaels) models exercises while the motions of the player are shown as a red or green shape on the right side of the screen. The color changes based on player performance, with green representing correct timing. A virtual Biggest Loser contestant also performs the exercises in the background. The camera is sensitive to the presence of others in the room. The red blob near the player’s foot is a cat, which has caused the game to interpret the player’s movement as incorrect (hence the red color and “follow the trainer”).

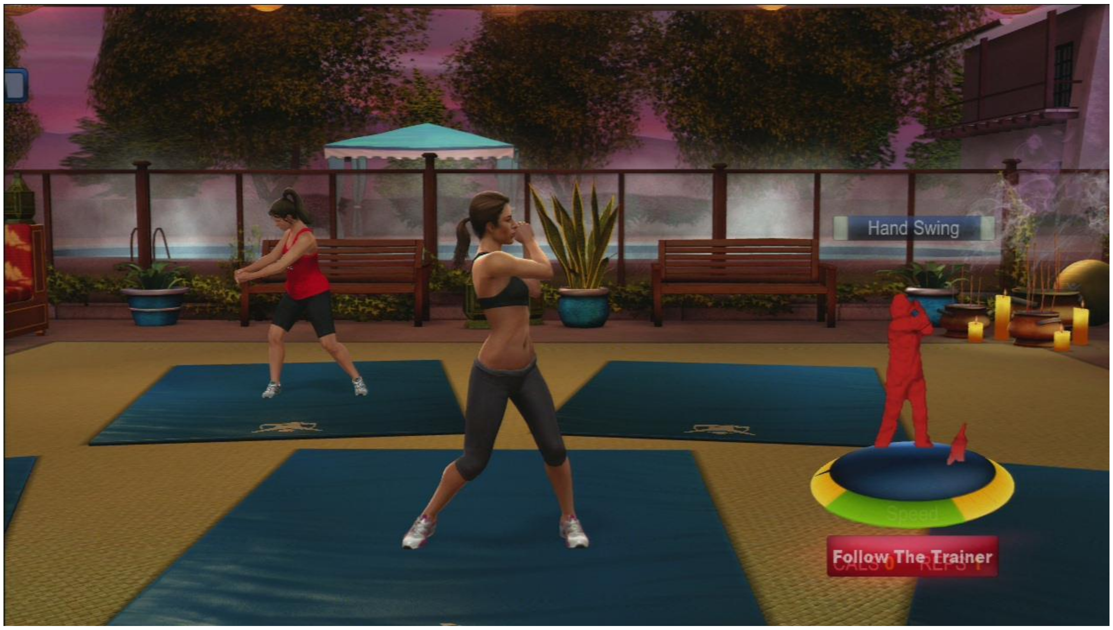

The game provides in-depth feedback on which parts of the exercise are being performed correctly. The green colors next to “Arm Swings” and “Squat Depth” as well as the green color of the player shape indicate that all parts of the exercise are being performed correctly.

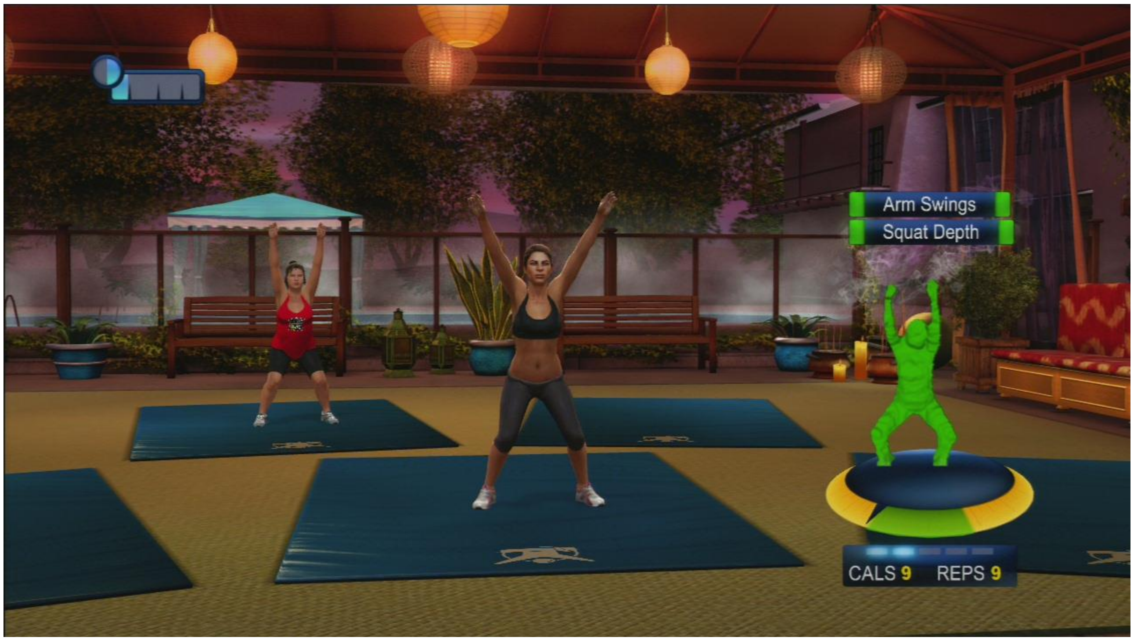

EA Sports Active 2

EA Sports Active 2 (released by Electronic Arts in 2010 for Wii, Playstation 3, and Xbox 360. The version used in this study was for the Xbox 360) uses both the Kinect camera-based controller and a dedicated peripheral heart rate monitor to evaluate player performance. The screenshot below shows the goals screen, where players can set specific goals and plan a schedule of workouts to reach their goals. The bottom of the screen for all menus shows the GamerTag, avatar that the player chose to represent her/himself, and current heart rate. (Note: the heart rate monitor was not worn for these screenshots, and thus no numbers are shown on screen.)

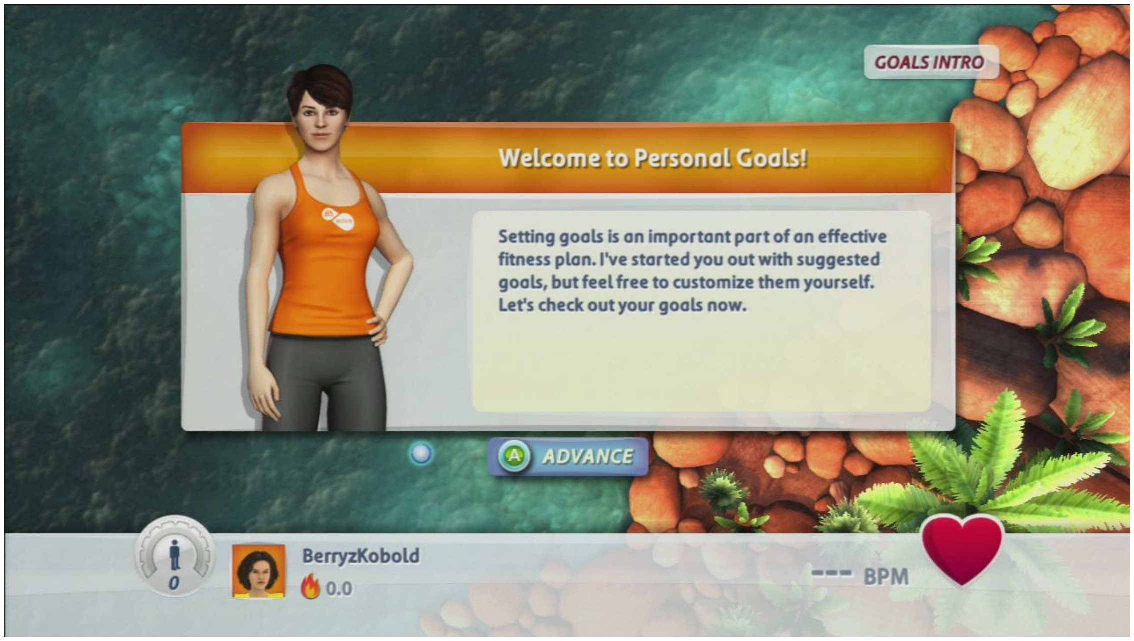

This screenshot shows current progress towards the chosen goal and the time limit for achieving the goal.

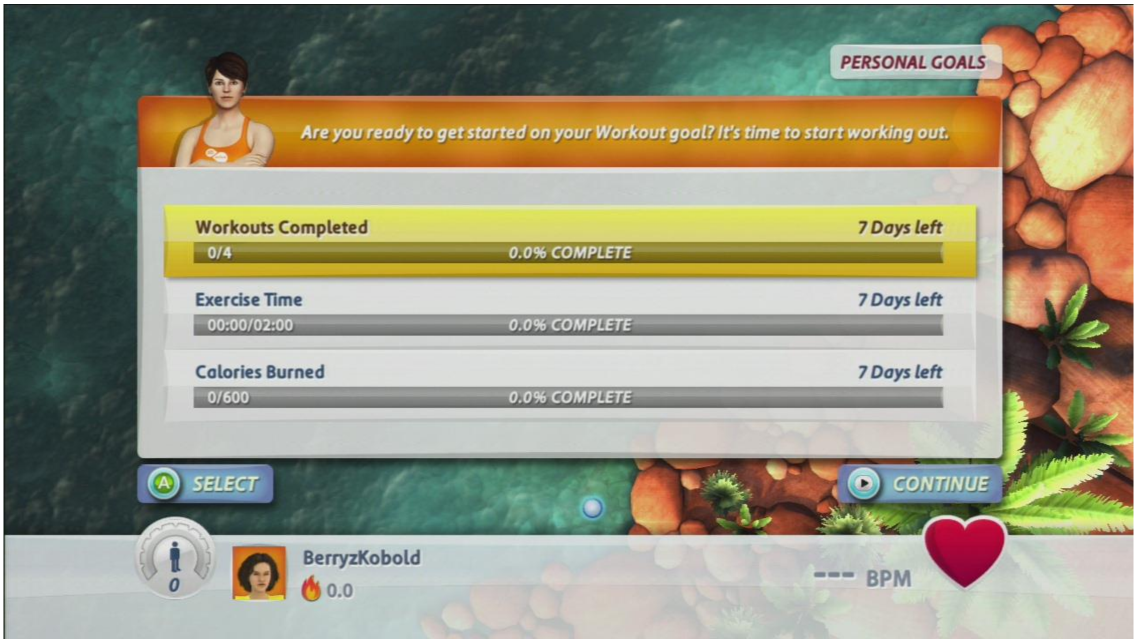

This screen is a typical workout screen from the game. Calories burned, time spent working out, repetitions of the current movement completed and total repetitions to be completed, and current heart rate are shown. The personal trainer (players can choose male or female trainers) models the movements. Players can move on once the game has detected that they have completed the number of movements required.

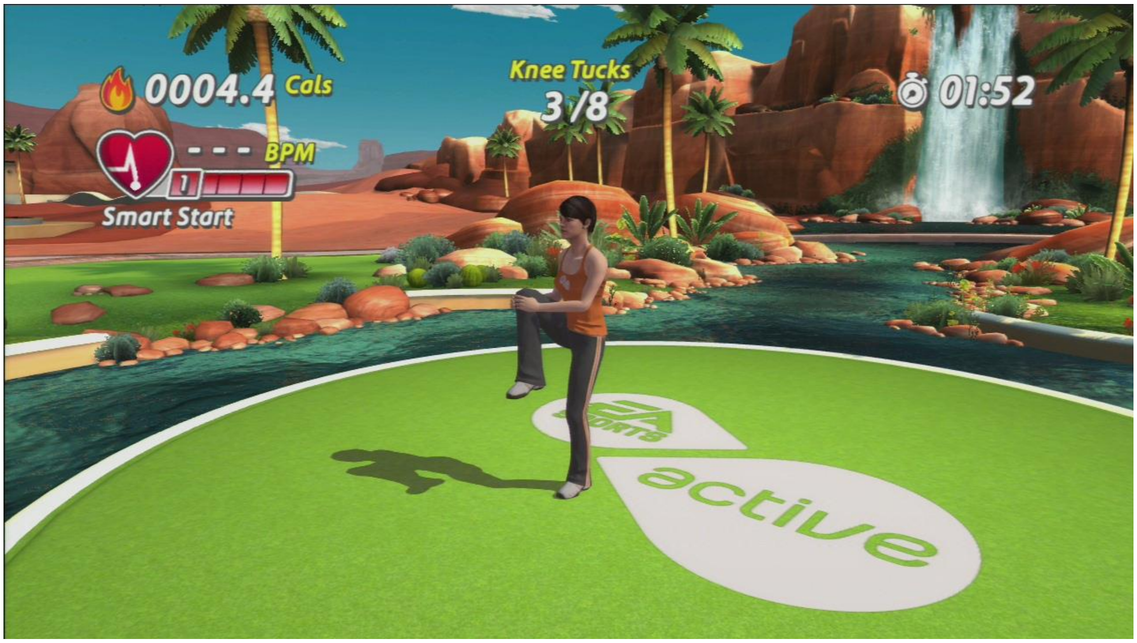
0004.4 Cals
Knee Tucks
3 /8
01:52
- - - BPM
1
Smart Start
active

Fit in Six

Fit in Six (released by Ubisoft in 2010 for the Wii and Playstation 3. The Playstation 3 version was used in this study) uses a combination of a camera-based controller (Playstation Eye) and handheld controllers (Playstation Move). The screenshot below shows how players are represented in the game. Real-time video of player movements is shown to the side of the virtual trainer who models the necessary movements. Time is shown on the bottom of the screen. The dial to the bottom right keeps track of how well the player is performing, and written feedback comes up on the screen at certain points in the workout ("good," etc.). The green light on the progress bar shows how far into the exercise players are at the time.

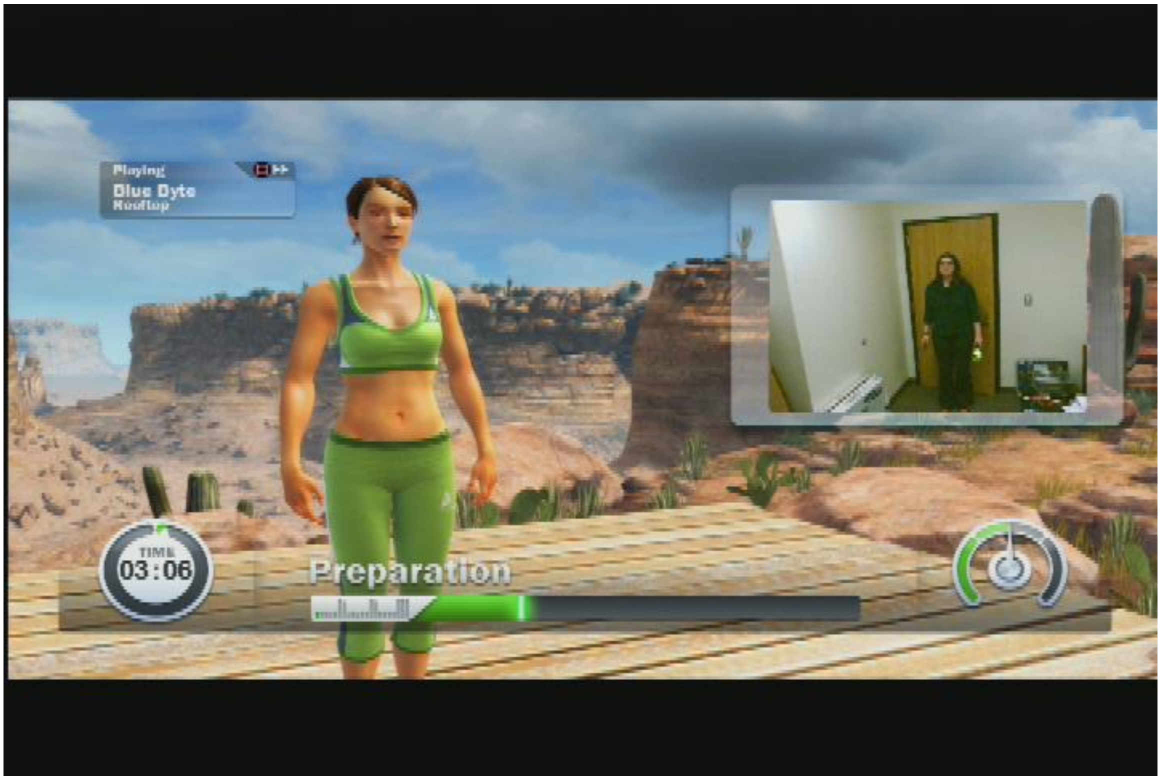

The next screenshot shows the player's chosen goal, the types of exercises needed to complete those goals (i.e., cardio and strength training to achieve the goal "burn off your extra energy"), and a choice of different exercises to perform. The menu shows the exercise duration and types of exercise involved (e.g., strength, cardio, flexibility).

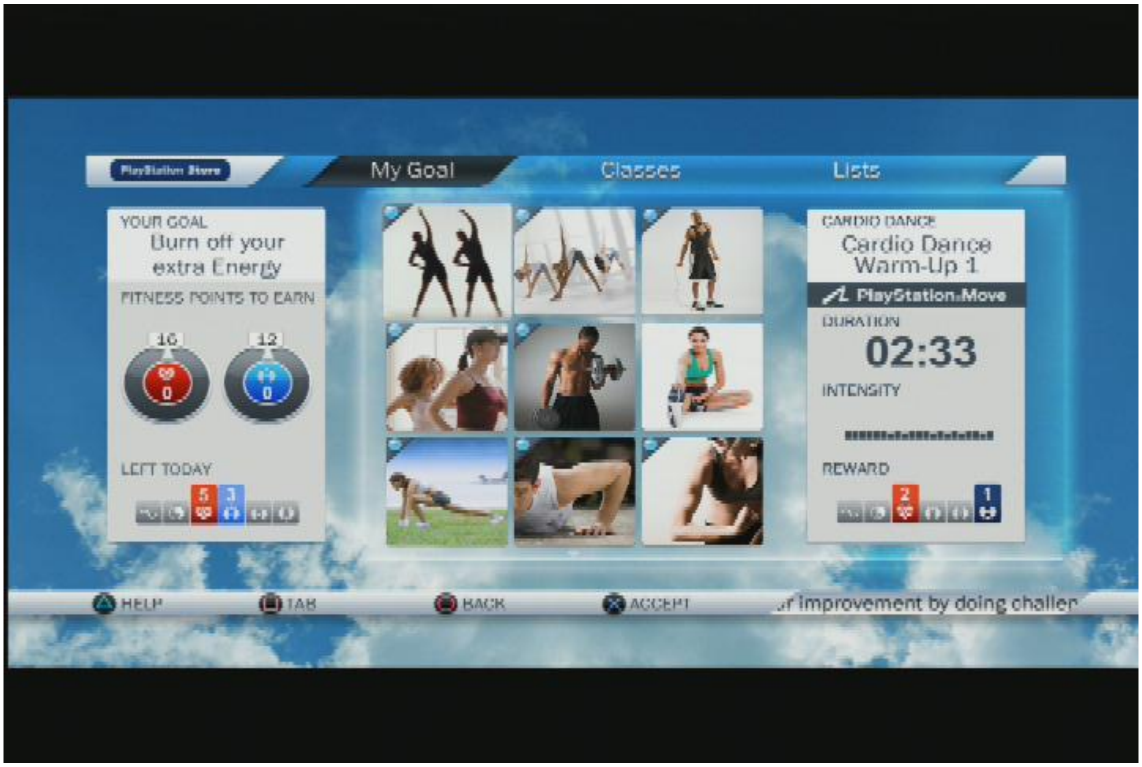

Here, the progress screen is shown. Progress is recorded as “Fitness Points” which are divided into six types of exercise and graphed to show player choices. There is a separate area that shows progress in specific “challenges,” which are difficult workouts.

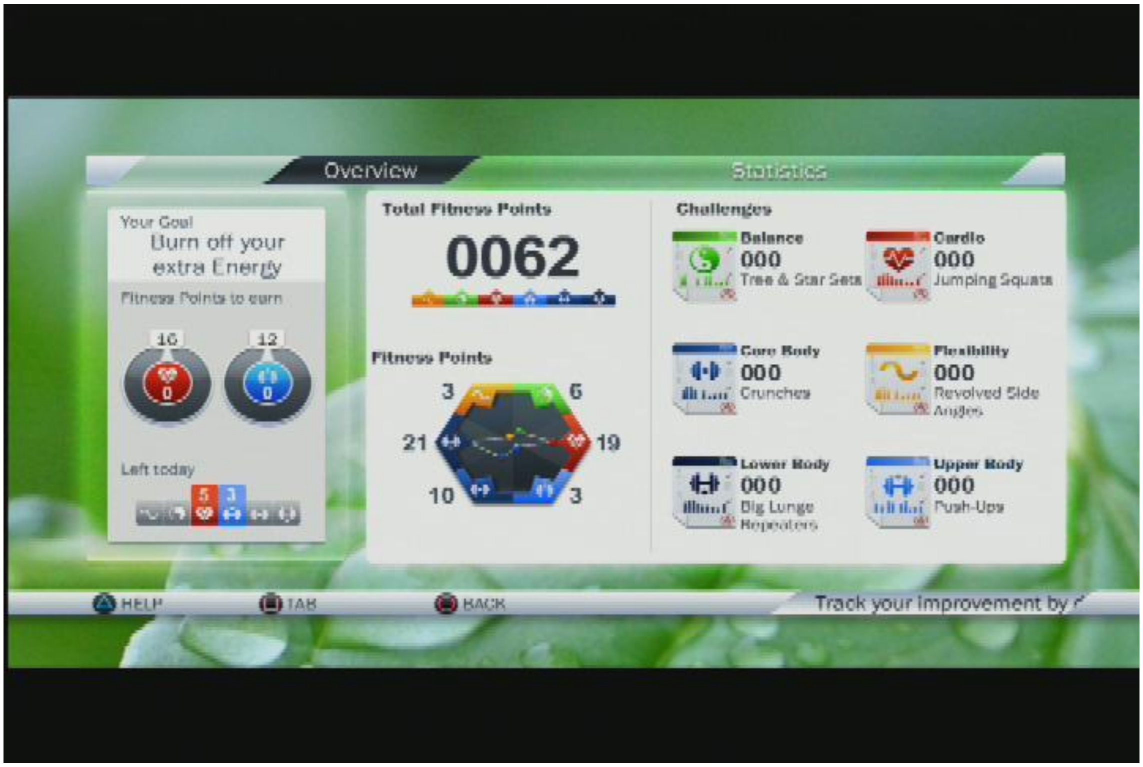

Get Fit With Mel B

Released by Deep Silver/Sony Computer Entertainment America for Playstation 3 in 2010, this game uses a combination of camera and handheld controllers similarly to Fit in Six. The first screen shot shows part of the process of choosing goals in this game. Players may choose two complementary goals and determine which is more important by assigning them weight. Goals come in a variety of types. Here, the player has chosen an equal balance between “looking good” and “weight loss” as the two goals of the game.

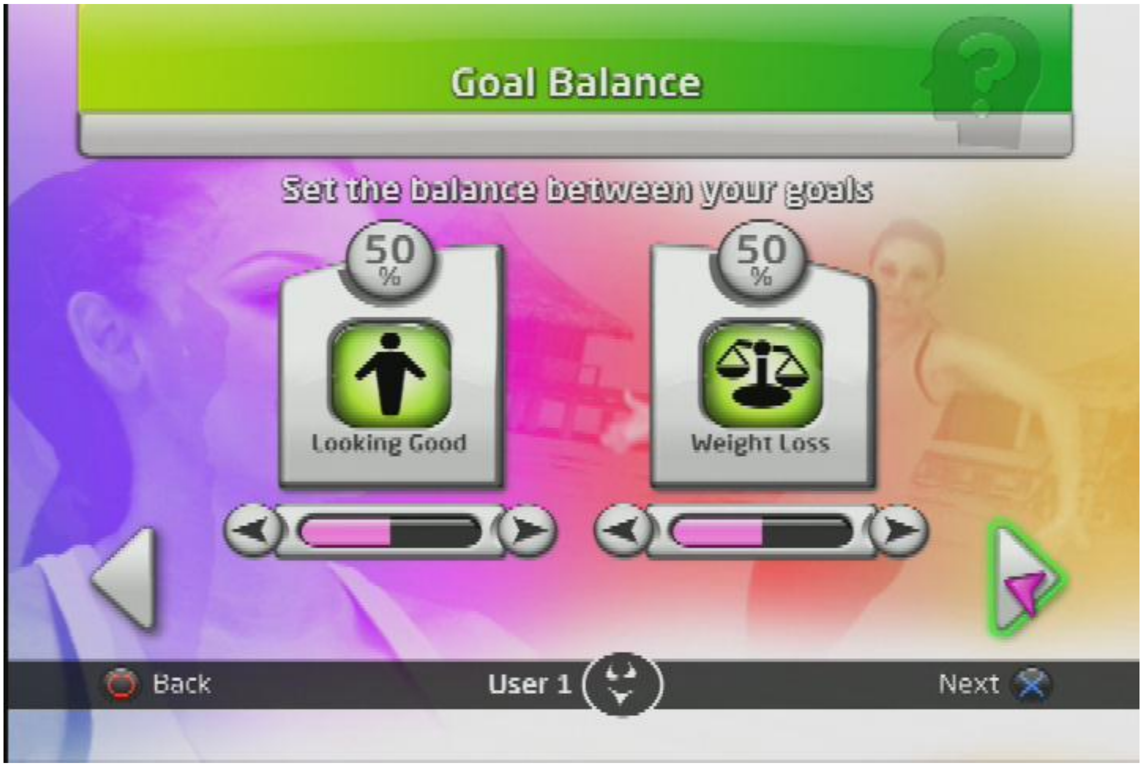

In contrast to many other fitness games, this game shows actual video of the celebrity trainer, Mel B, modeling the exercises (as opposed to a computer-generated representation of her). Time, the model’s completed repetitions, and the player’s completed repetitions are shown at the top of the screen. Players can press a button to enter a tutorial (guided practice), where scores are not kept and they can practice until they feel more comfortable with the exercise. The green bar underneath “You” at the top shows how accurately the player is performing each movement, based on the camera and the handheld controller. The more full the bar, the better the player is performing.

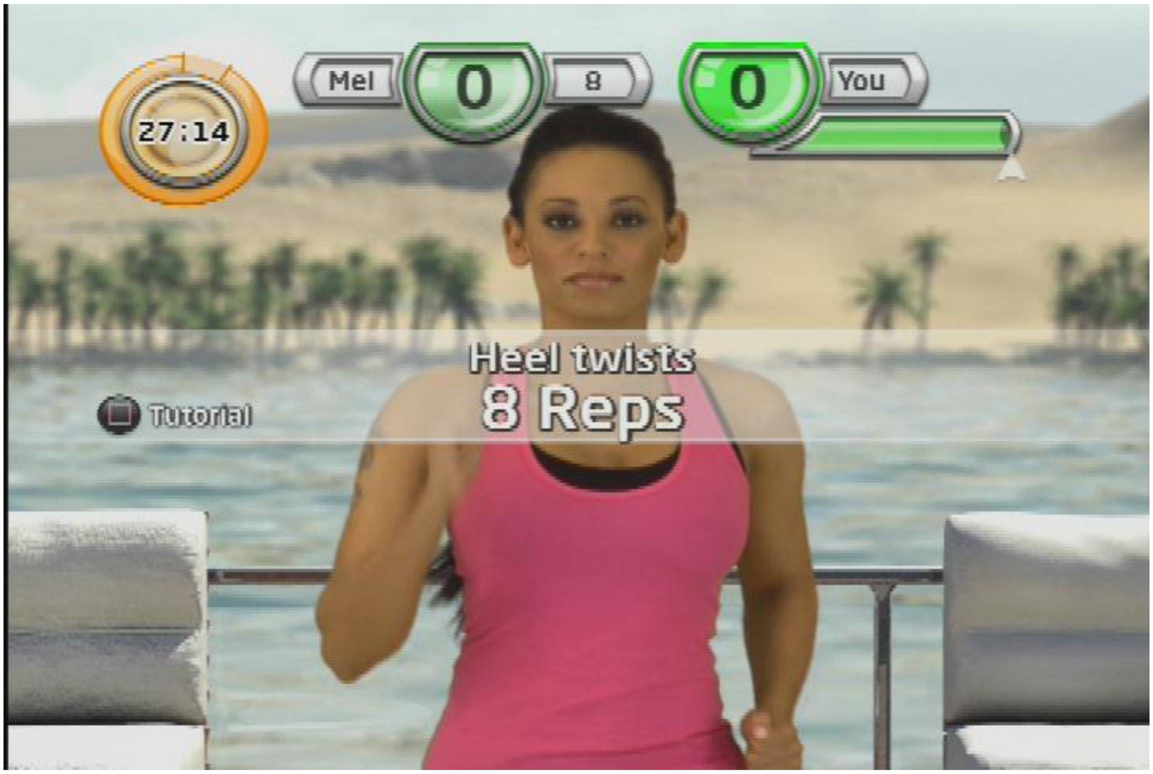

Real-time video of the player can be turned on or off, depending on player preference. The player's video is shown to the right of the trainer so that players can compare their movements directly to hers. The screenshot below was taken during a warm-up period, so no player feedback was provided at the top of the screen.

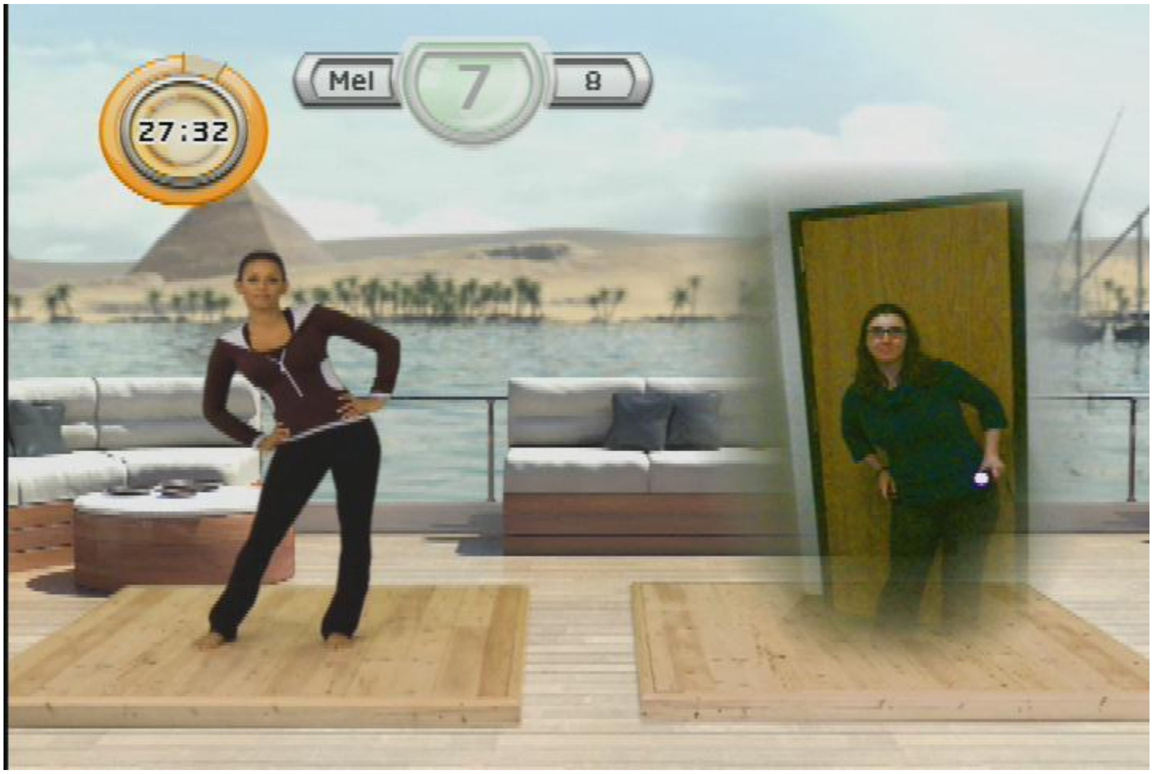

Jillian Michaels Fitness Adventure

Jillian Michaels Fitness Adventure (Majesco, 2011 for Xbox 360) differs from many other fitness games in its explicit attempt to incorporate traditional video game elements into workouts. Although traditional workout sessions are available, a large portion of the game is spent in a "fitness adventure" that closely resembles minigames from more party-themed Kinect games. Players run in place as their character explores a virtual environment. Running speed influences the speed at which the player travels through the environment. A minigame is incorporated into the running such that players must reach out to collect green circles but must avoid red circles. Intermittently, obstacles and traps appear that players must use exercise motions to overcome. For example, in the screenshot below the player must perform 10 "water pumps" to pull the lever and open the door. The game shows a computer graphics representation of Jillian Michaels, a celebrity trainer, in the left bottom corner. In the bottom right corner, real time video of the player is shown. The video changes colors based on performance, and a green circle appears when moves are performed correctly. At the top of the screen, the number of repetitions completed and necessary are shown as well as the name of the exercise and the time spent in the level.

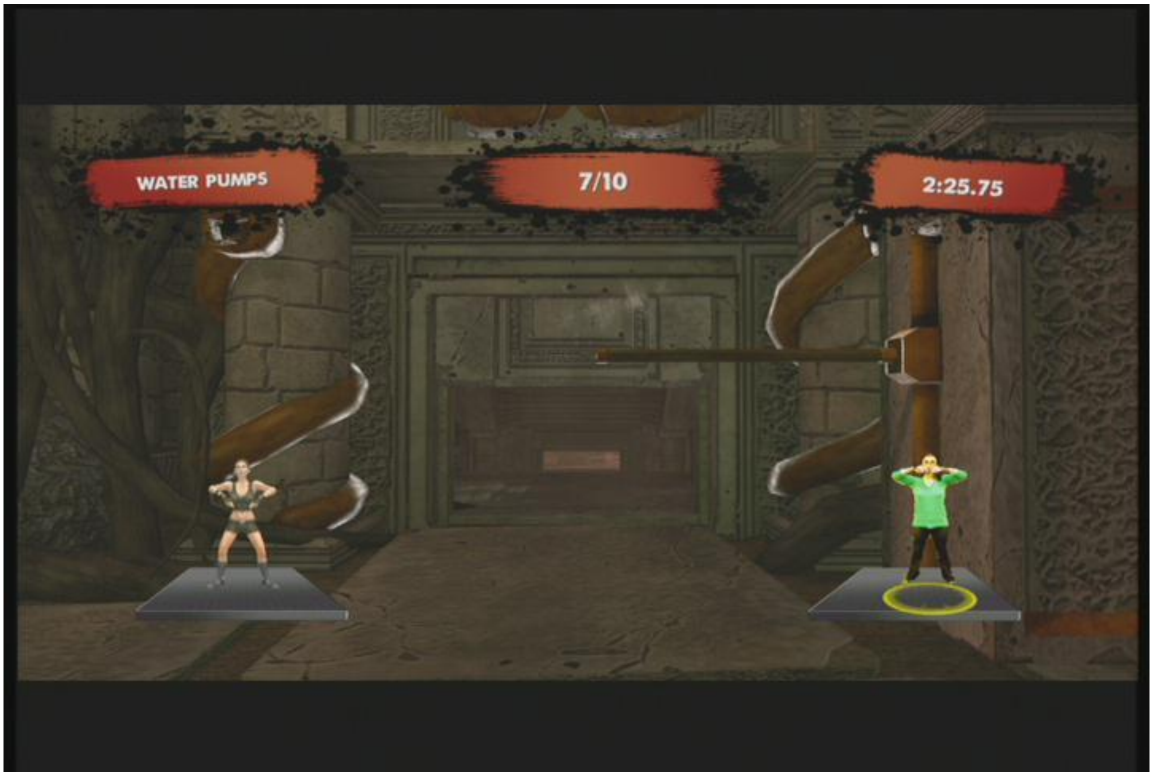

At the conclusion of workouts, or "levels," players are given feedback. The number of each type of circle obtained is shown as well as calories burned, time spent, and accuracy percentage broken out by each type of exercise performed.

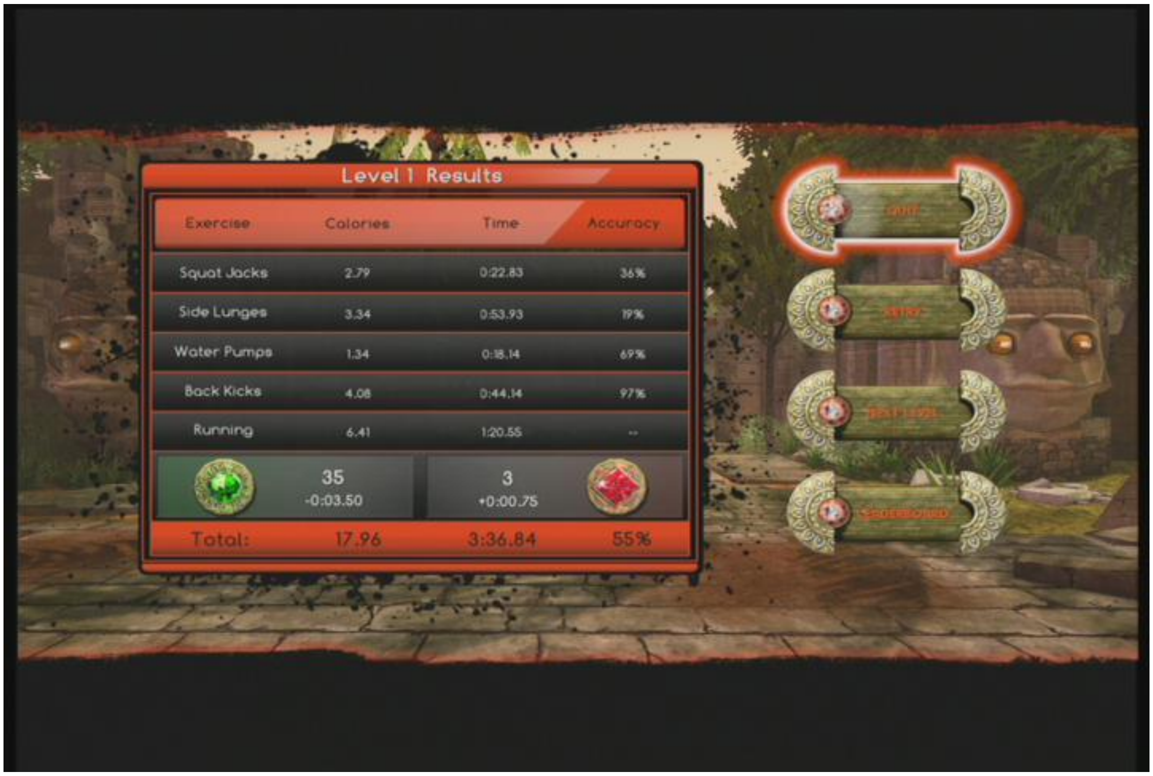

The game offers a workout calendar that marks days the game was played. It also contains an online leaderboard that ranks players from Xbox Live.

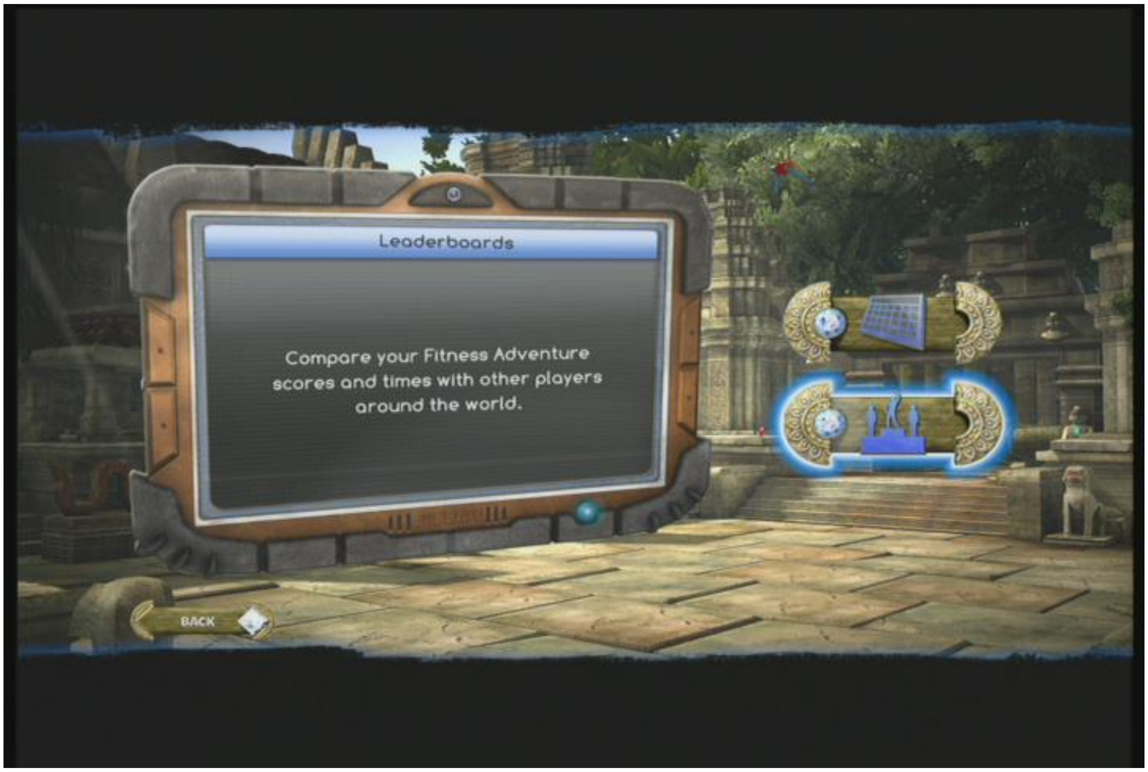

The Fight: Lights Out Fitness Pack

The Fight: Lights Out was released by Sony Computer Entertainment America is 2010 for the Playstation 3. The downloadable content Fitness Pack was released on the Playstation Network in 2011. The Fight is a fighting game that follows the player character as he undertakes a career in illegal underground fighting. The game uses a Playstation Move controller in each hand as well as the Eye camera to evaluate punching and kicking motions. The Fitness Pack adds on behavioral strategies to the game, achieving a sufficient fitness focus for inclusion in this study. It also adds fitness-specific minigames such as lunges, arm circles, etc. The screen shot below shows the fitness stats page, where players can see a breakdown of their calories burned in different types of exercises in the game. They can also see graphs of their score and calorie burn over time.

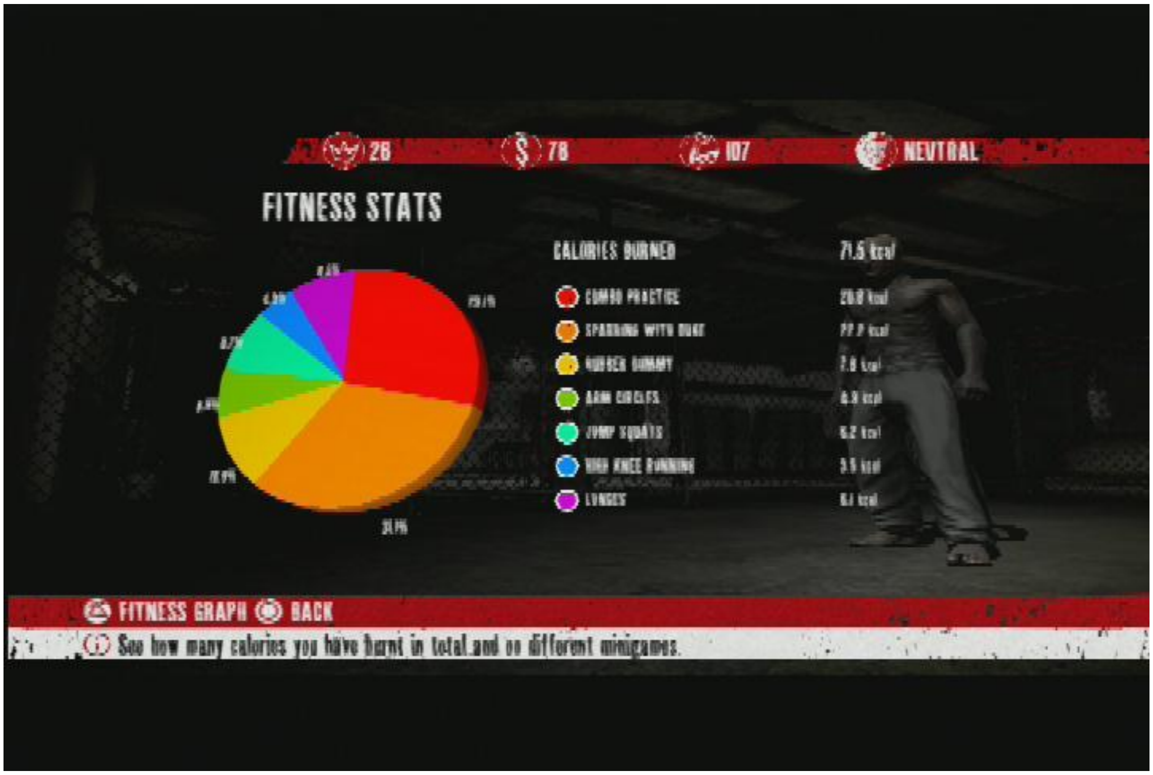

The screenshot below shows a sparring match fitness minigame, during which players are to punch the mitts held up by the trainer. The bar at the top of the screen shows time, score, and calories burned thus far in this specific minigame. This game includes a celebrity character (Danny Trejo) as the trainer.

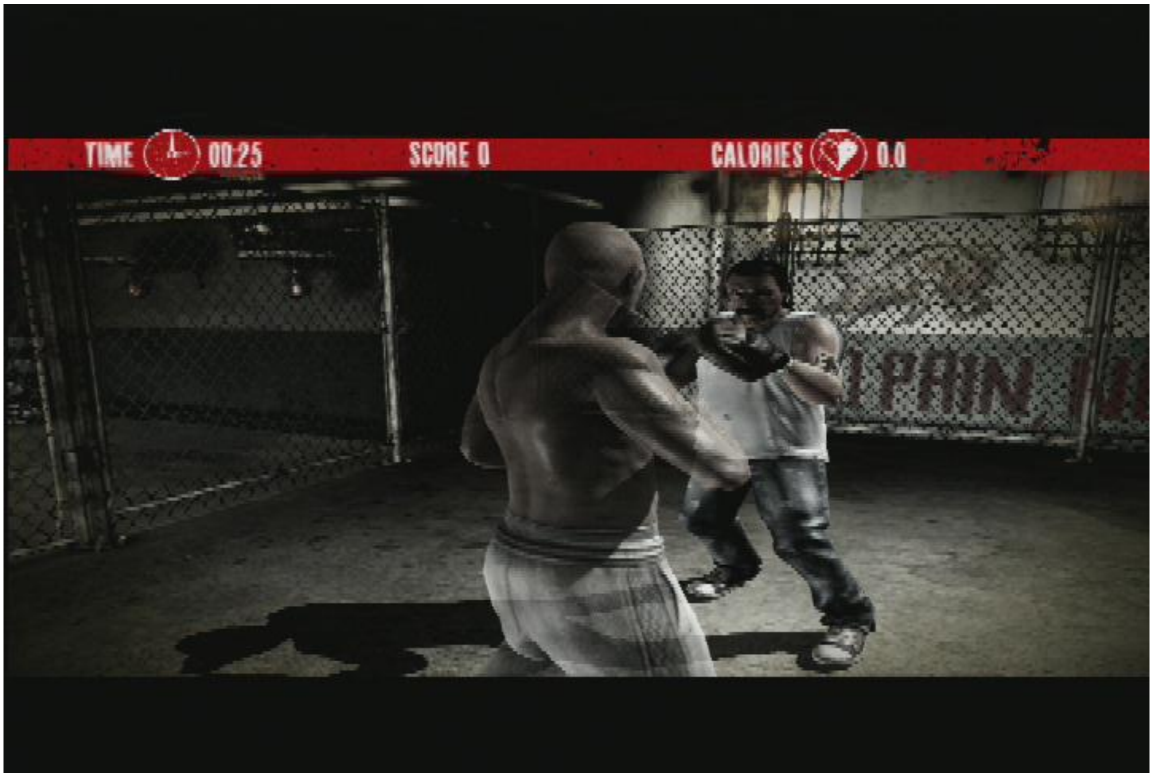

The training results screen provides players with feedback on their performance in each fitness minigame. Skill points can be redeemed as rewards in other parts of the game.

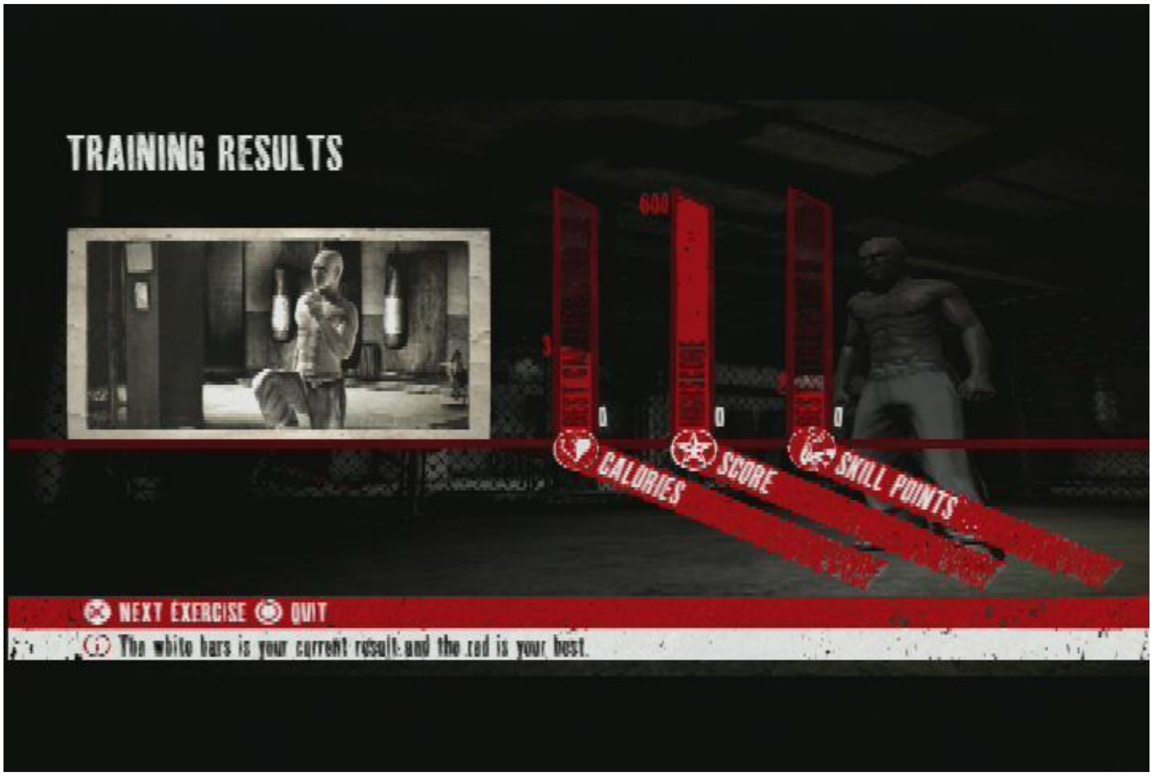

UFC Personal Trainer

UFC Personal Trainer (THQ, 2011, released for Wii, Playstation 3, and Xbox 360 – Xbox 360 version used here) is an in-depth workout game that advertises its collaboration with the National Academy of Sports Medicine in creating the game. It uses celebrity fighters from the Ultimate Fighting Championship to model mixed martial arts training for fitness. The feedback provided by this game is extensive. The screenshot below shows the different trackers included in the game, including personal information (input by the user), a basal metabolic rate calculator, a workout journal, rewards, and graphs. Graphs are available for several different variables, such as calories burned and weight. The green shape at the bottom right of the screen is the shape of the player, moving in real time, from the camera controller. The microphone at the bottom left indicates that players can use voice commands to control the game.

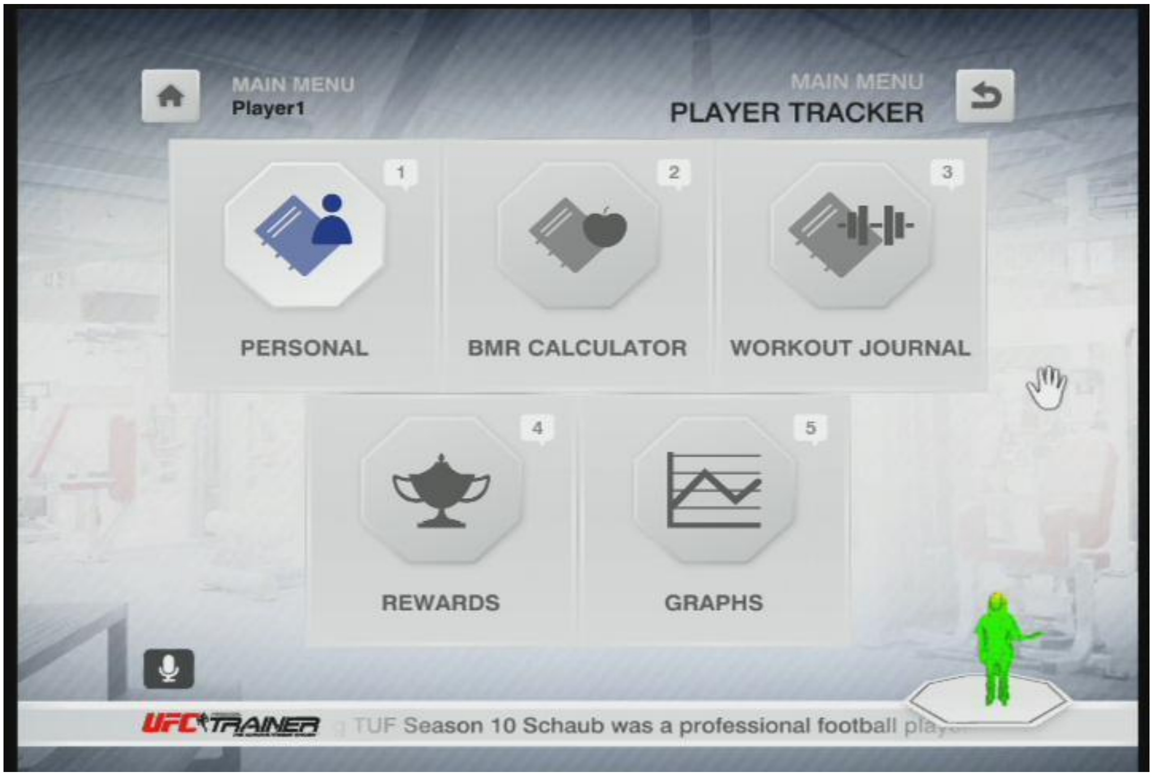

The workout journal tracks total time spent playing the game, average time spent per session, total calories burned, average per session, and shows the type of exercises that the player has been performing (cardio, strength).

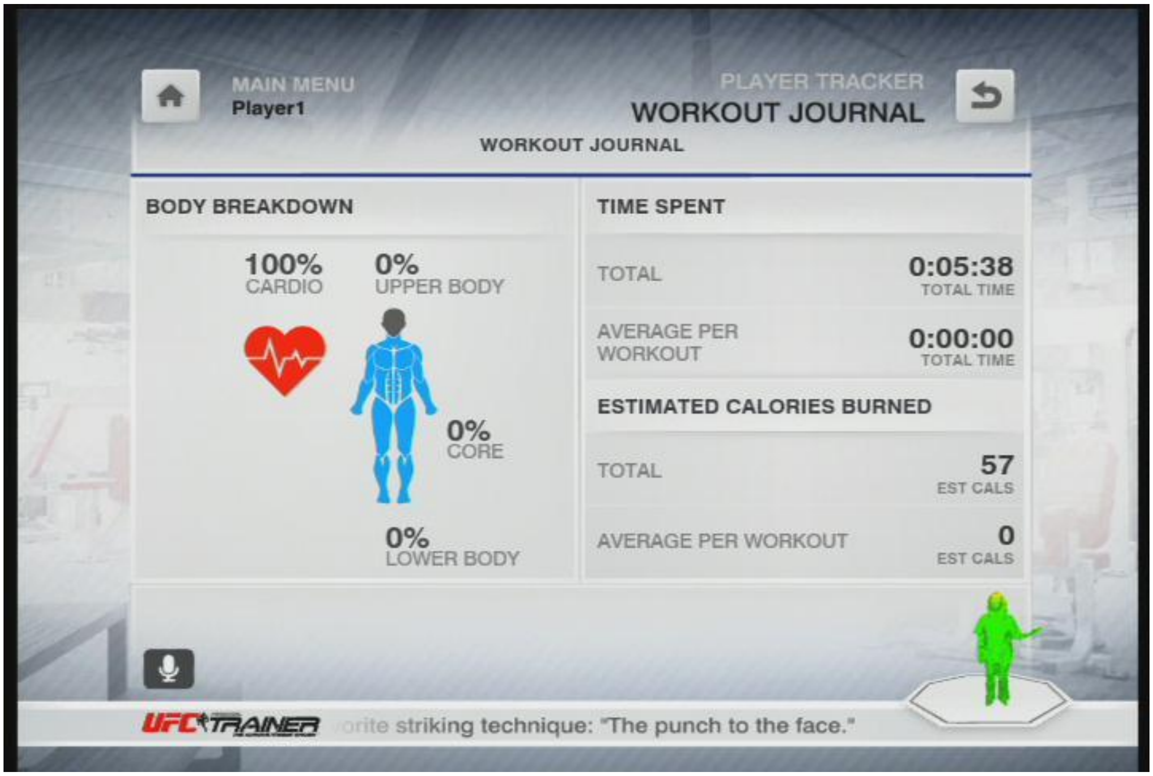

Exercises are performed with UFC fighters as models and trainers. In this screenshot, the player (shown in green at the bottom right) achieved a streak of correctly-timed punches. This streak was rewarded with an "achievement," which is a console-wide reward on the Xbox360 that all of the player's friends can see. This screen shows time elapsed in the specific exercise, score, and accuracy (the circle labeled 100). Players can also view their shape and compare it to the trainer when he is modeling exercises.

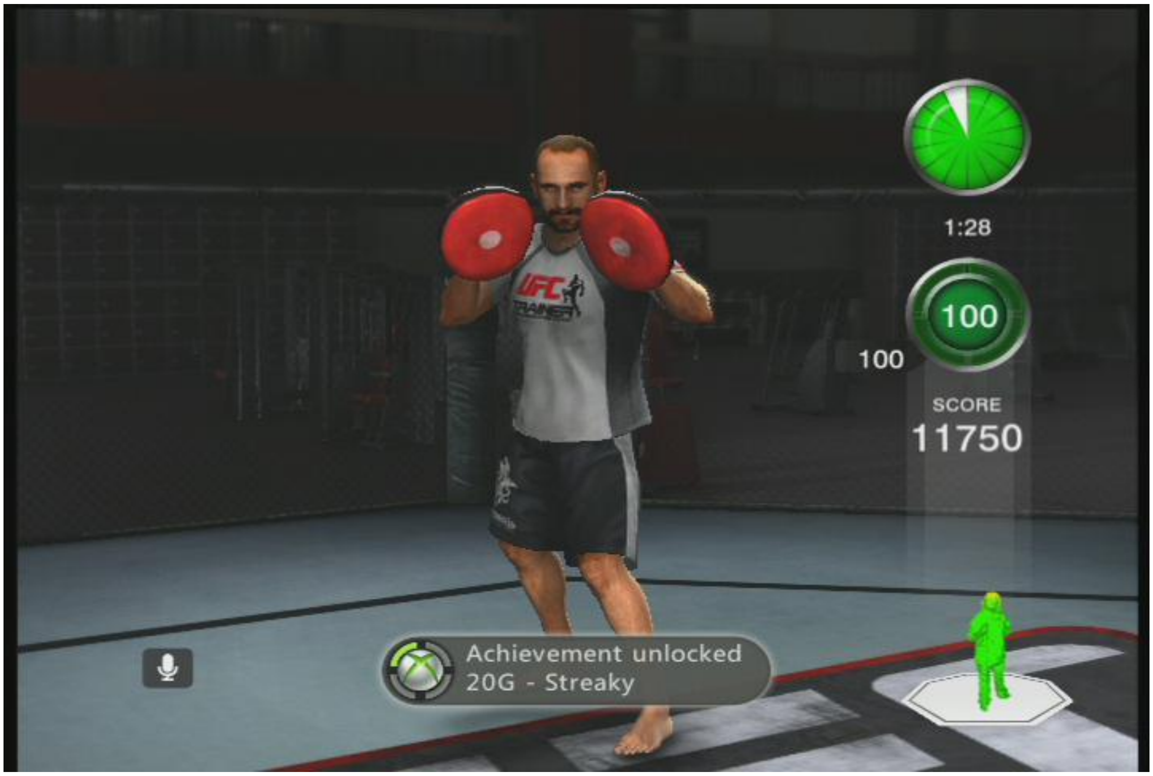

Your Shape: Fitness Evolved 2012

Your Shape: Fitness Evolved 2012 (Ubisoft, 2011, Xbox 360) is part of the Your Shape series of games. This iteration uses the Kinect camera-based controller to evaluate player movement and show real-time video of players as they exercise (a screenshot showing the video is provided in the manuscript text). Below is the goal-setting screen, showing player progress towards the goal “boost my cardio.” Also on the screen are the goal duration, frequency, and time limit. At the top right of all screens is the calorie bar, which shows total calories that the player has burned thus far in the game. The bar indicates progress towards the next calorie level.

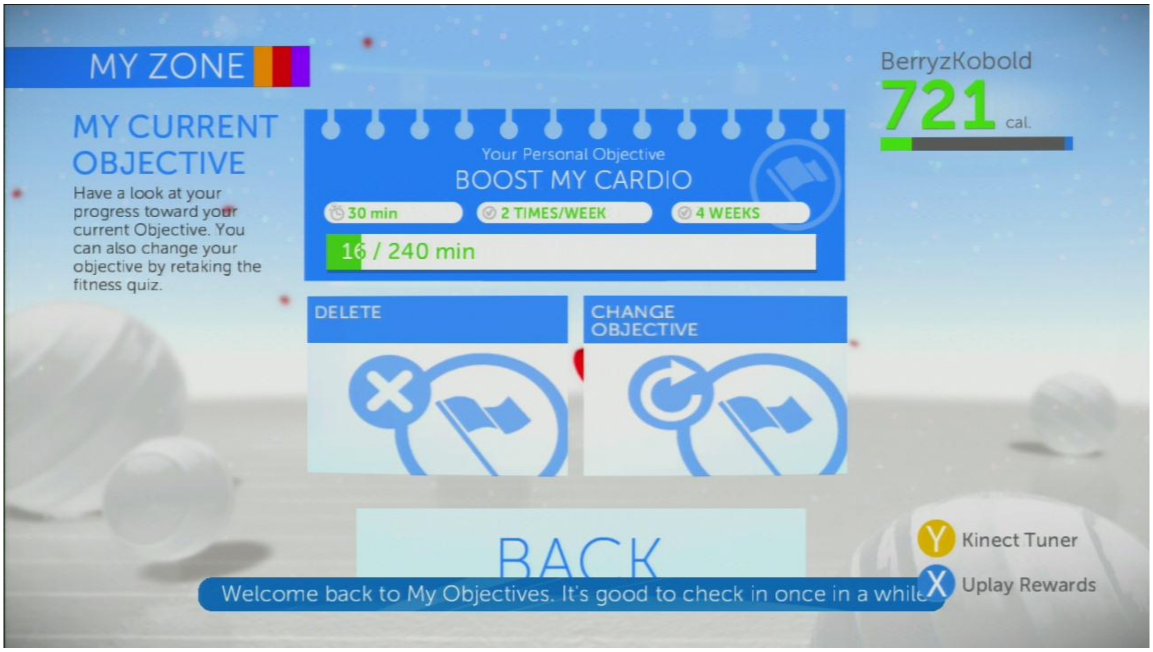

The calendar indicates when the game has been played with green stars.

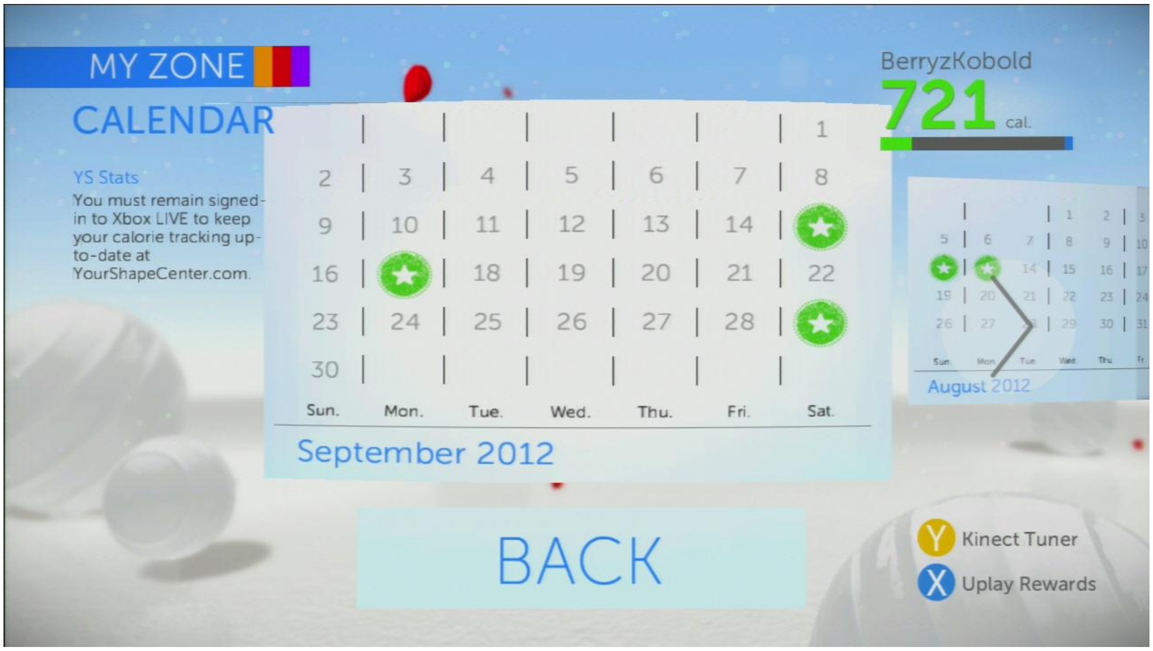

This screenshot shows choices of different classes within the game. In addition to minigames, the game includes dance and workout classes (e.g., boot camp, cardio boxing). Here, the “Zen energy” class options are shown, where players can choose different guided practice sessions before enacting the dances as part of a mastery experience.

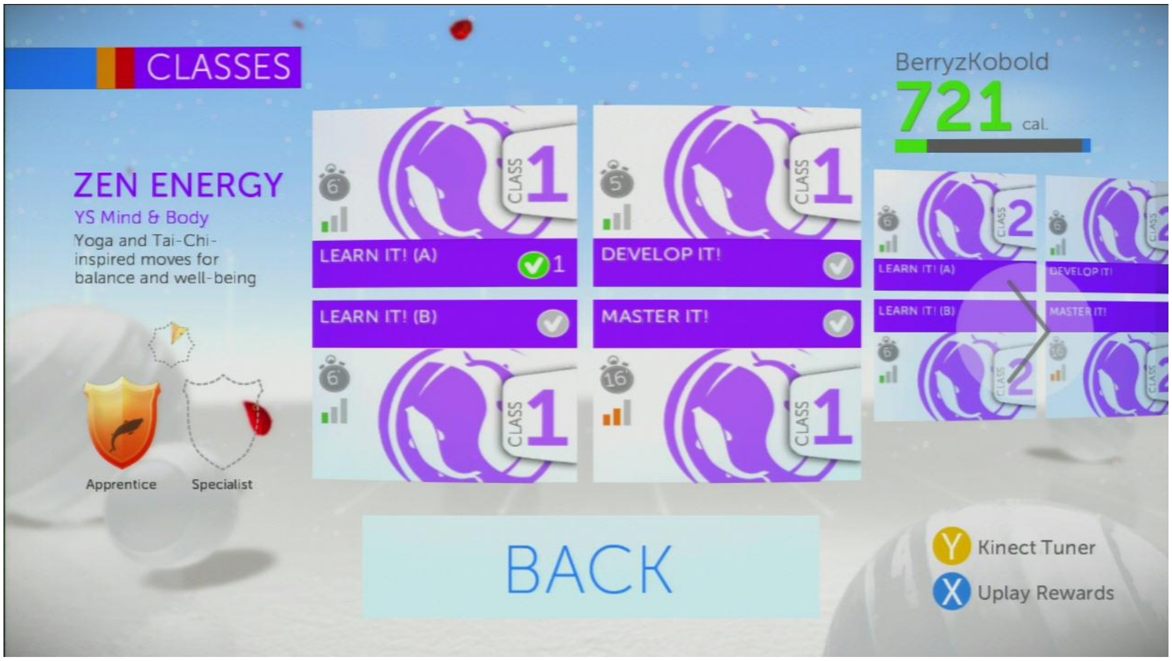
CLASSES
BerryzKobold
721 cal.
ZEN ENERGY
YS Mind & Body
Yoga and Tai-Chi-inspired moves for balance and well-being
Apprentice
Specialist
CLASS 1
LEARN IT! (A)
DEVELOP IT!
LEARN IT! (B)
MASTER IT!
CLASS 2
BACK
Kinect Tuner
Uplay Rewards

Zumba Fitness Rush

Zumba Fitness Rush (Majesco, 2012, Xbox 360) is part of the Zumba Fitness series of video games. It uses the Kinect camera-based controller to evaluate player movements. It includes real-time video of players as a shape in the bottom left corner. This game is focused on dancing for fitness. The screenshot below shows a guided practice session. The model shows players how to do a dance move at a slow speed, then speeds up as the player masters the move.

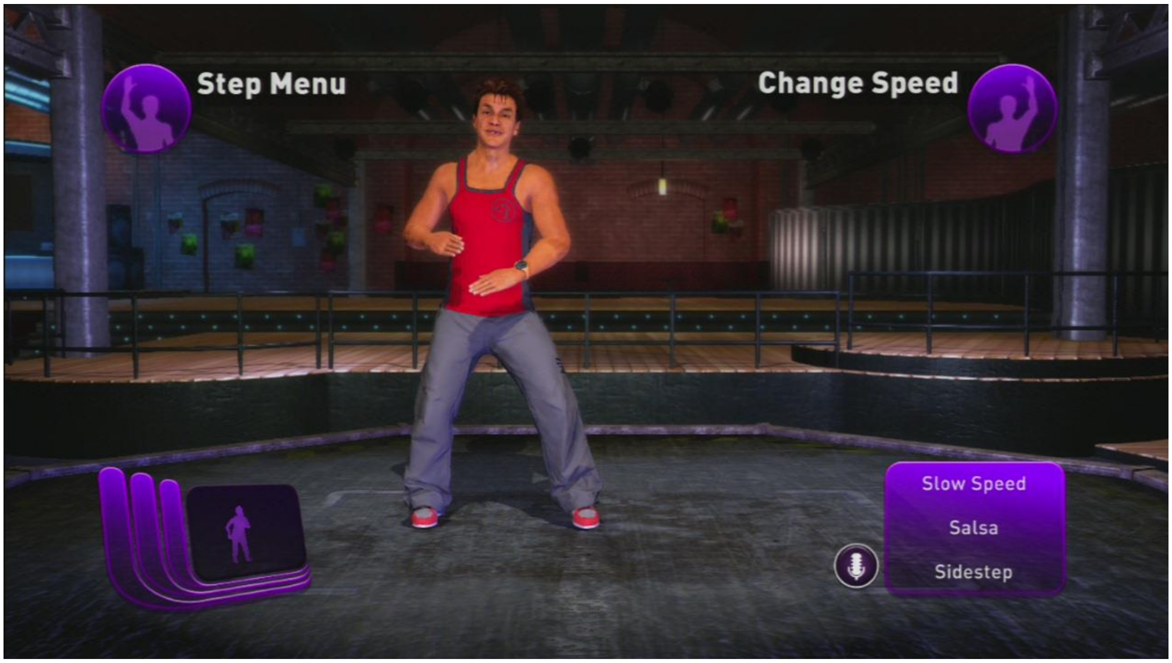

Below are some of the achievements that can be attained as part of this game. This screen can be viewed by others who have friended the player on Xbox Live, the online multiplayer and entertainment service of the Xbox 360. The achievement shown here, "Zumba Diploma," was earned after earning all of the achievements for completing each guided practice/tutorial session.

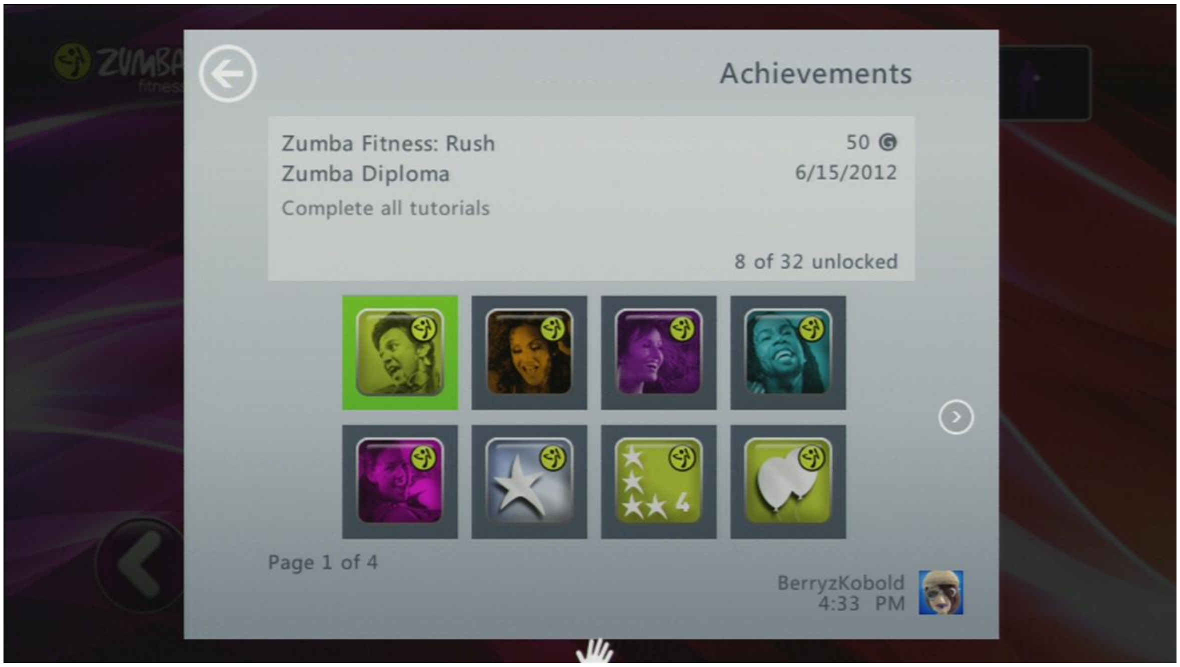

Gameplay is similar to other fitness and dance-oriented games. A model demonstrates movements while players mirror those movements at home. Animated boxes (the pink box to the right in the screenshot below) appear prior to a new move to let the player know what kind of dance is coming up next. The stars at the bottom indicate how well the player has performed. The box at the bottom left shows the players movements in real time, and the bars next to it light up as moves are performed correctly.

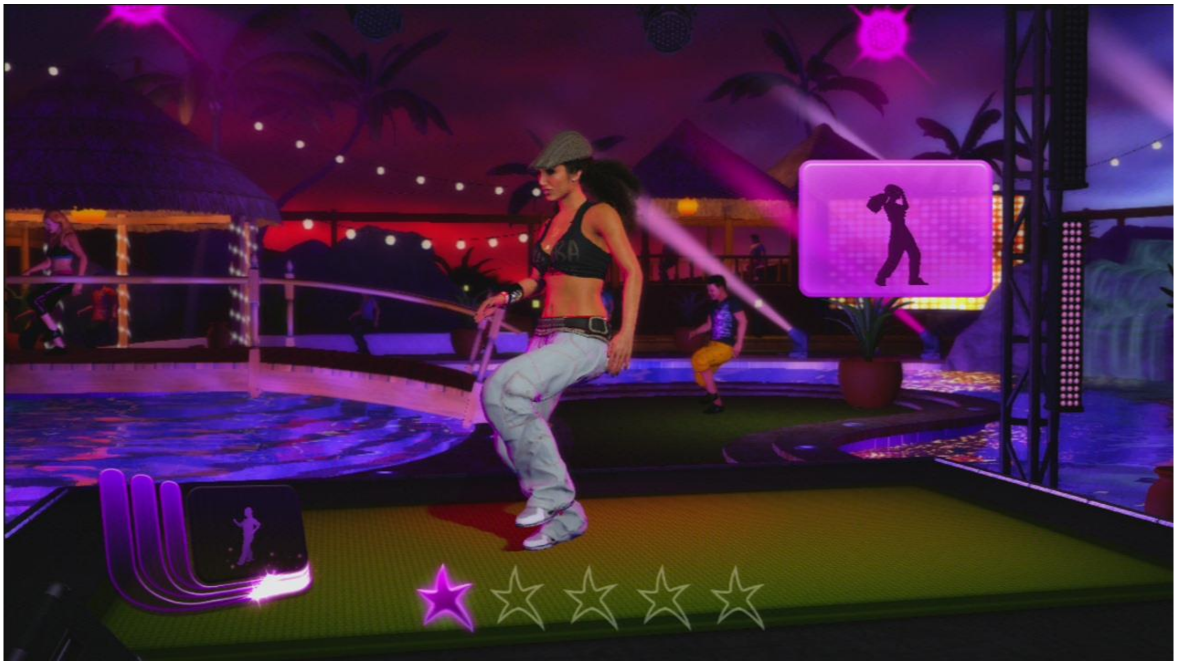

Non–camera-based games

10 Minute Solution

10 Minute Solution (Activision, 2010, Wii) is a video game based on a popular series of workout videos. The game allows players to choose from several types of exercises mostly centered around boxing and aerobics.

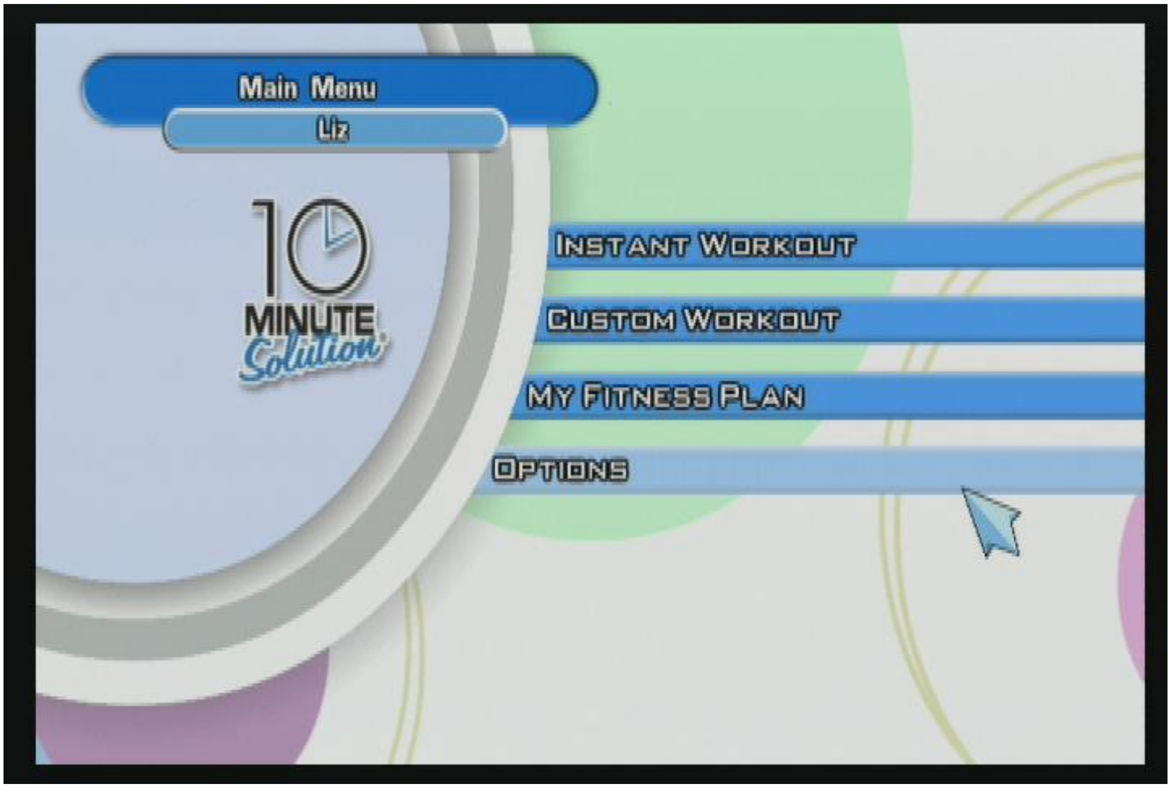

This screen shows the options for playing the game. Players may also choose to watch an exercise video without having their performance evaluated by the game.

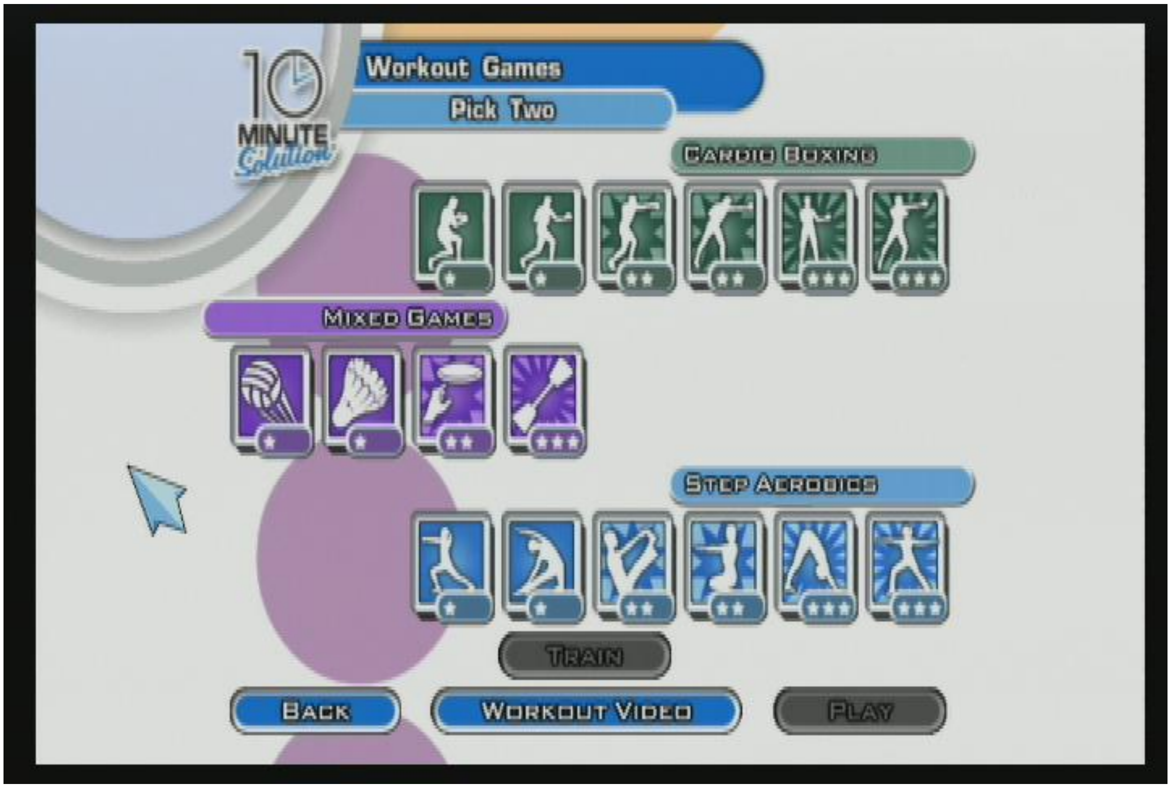

Pictures travel from the bottom of the screen that indicate what the player is supposed to do. When the picture (here, "cross") reaches the blue box, the player is to perform that movement. Their movement will be picked up by the hand-held motion-sensing controller and their avatar will mimic their behavior.

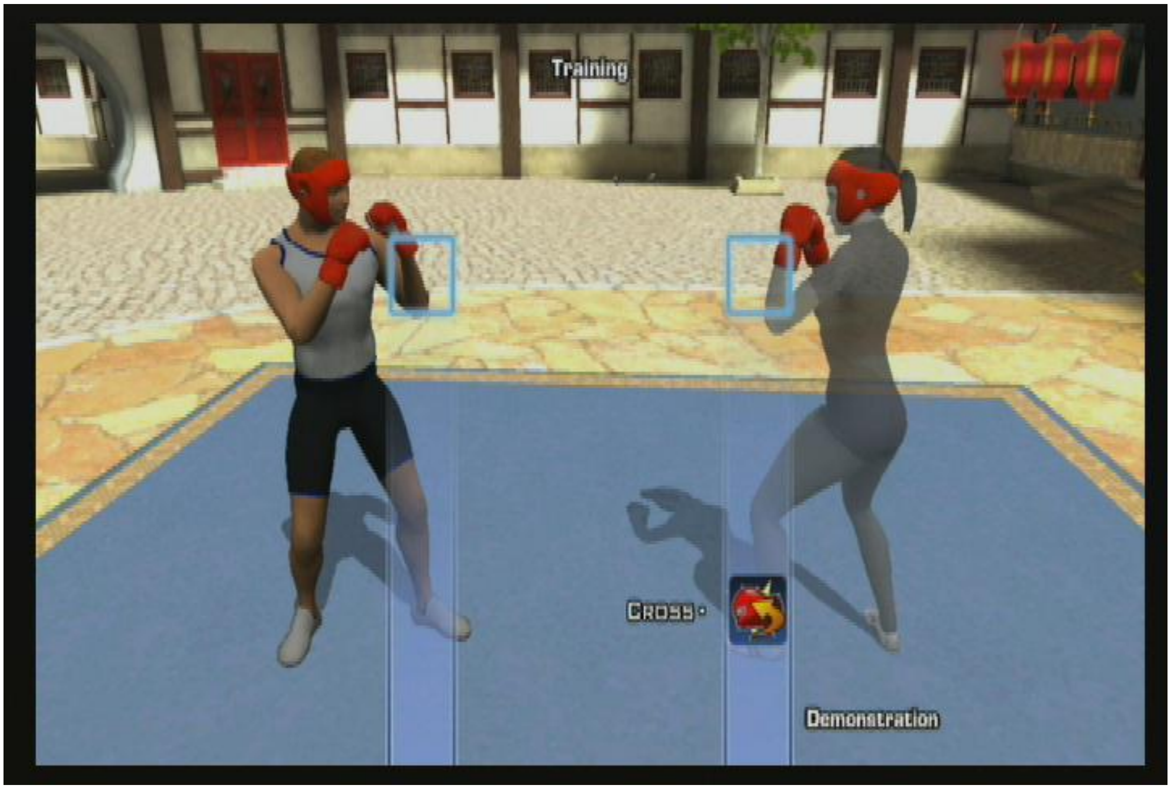

Daisy Fuentes Pilates

Daisy Fuentes Pilates (Sega of America, 2010, Wii) specifically concentrates its gameplay to Pilates exercises. Players can choose exercises, get tips on diet and exercise, and choose different outfits for the model to wear. There is a video introduction that includes celebrity Daisy Fuentes, but in the game itself she is represented by a computer generated character.

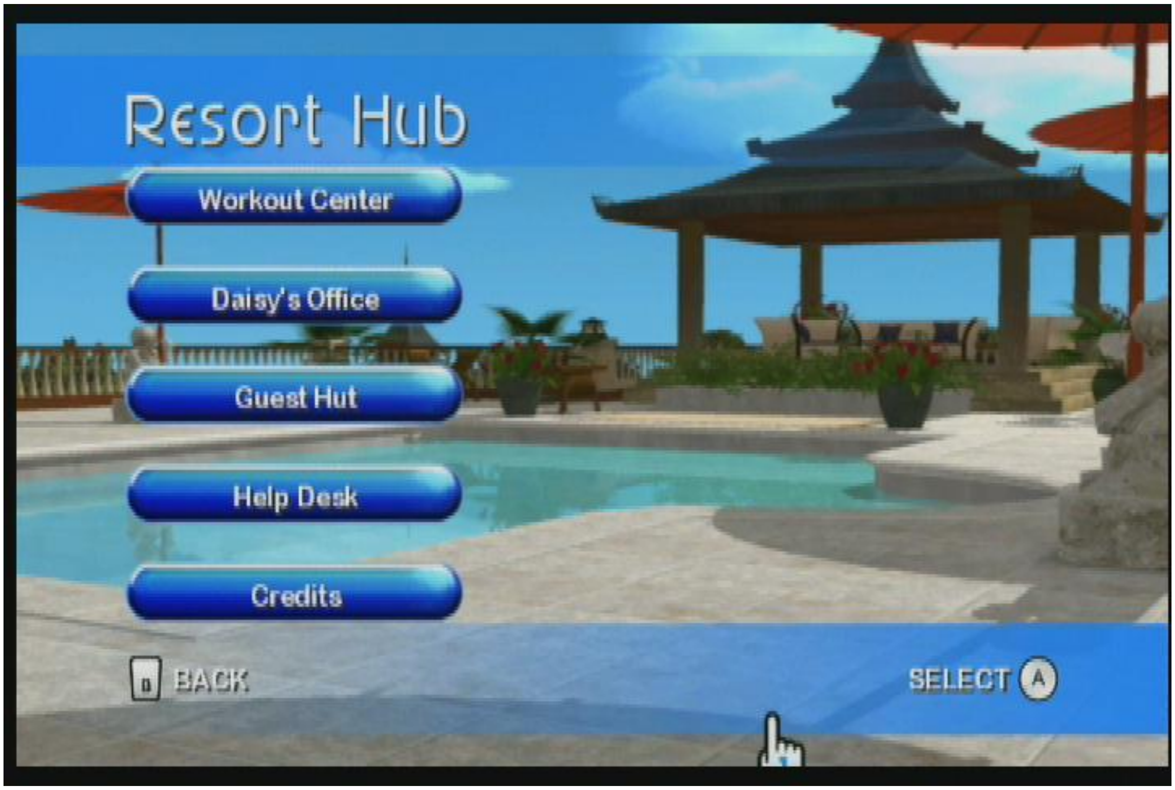

Players can watch the trainer model the exercise, engage in guided practice as part of the tutorial, or enact the exercise while the game evaluates their accuracy using motion controllers (handheld and balance board).

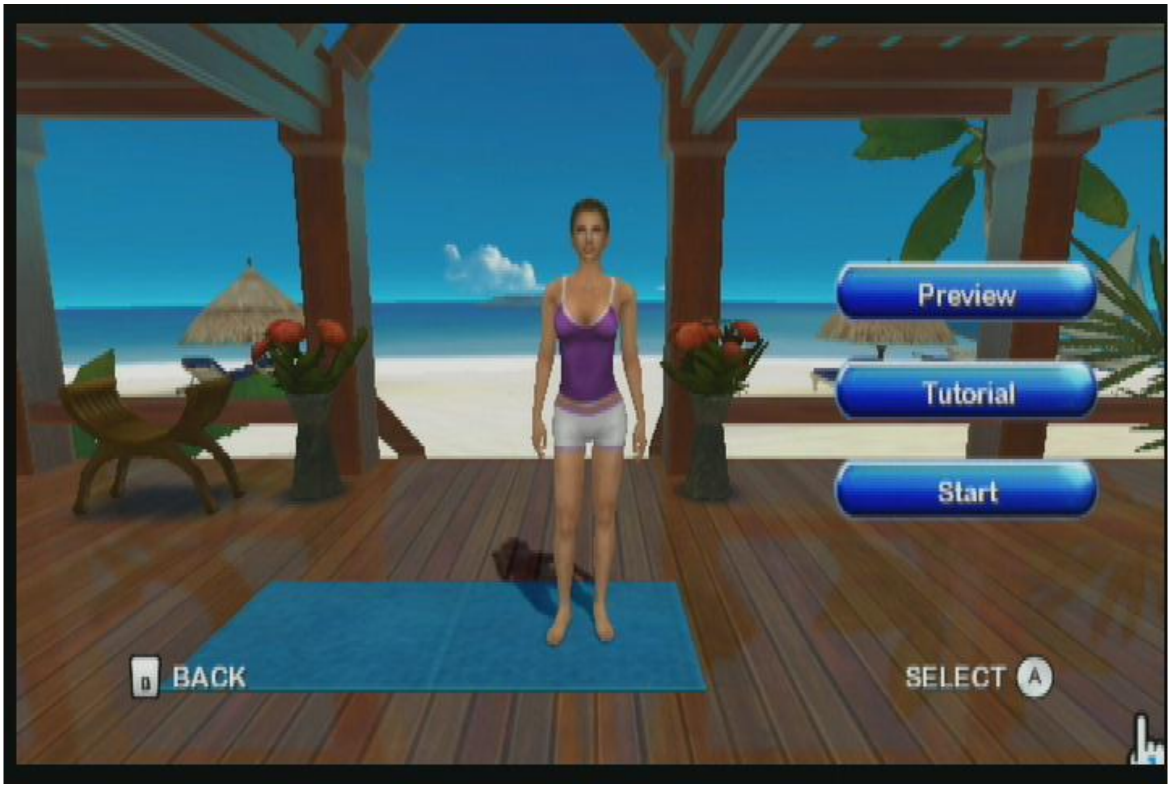

As the model performs the exercises, the number of repetitions and difficulty level are shown at the bottom of the screen. The model shows how to position the balance board so that it can evaluate the movement.

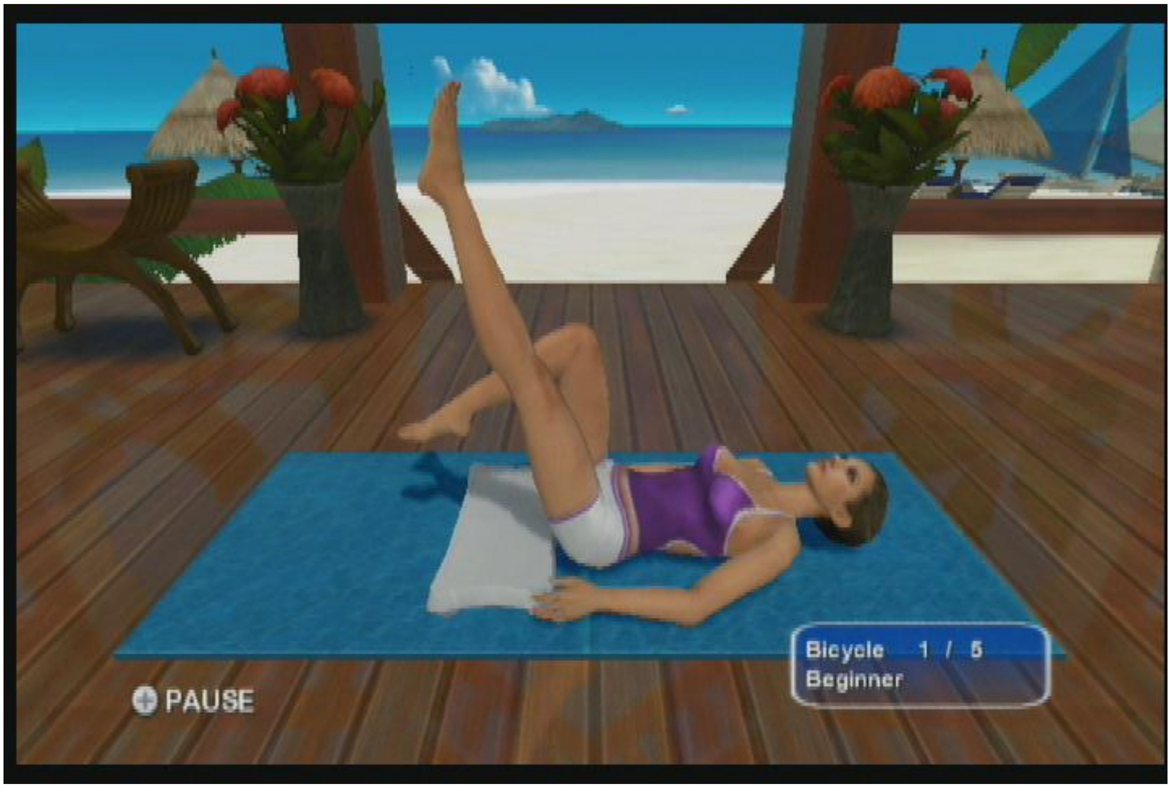

Exerbeat

Exerbeat (released by Namco in 2011 for the Wii) is a dance-themed exercise game with additional minigames. It includes strategies such as a calendar and multiplayer. Players use a handheld controller in each hand and perform a series of aerobic sessions. A trainer models the movements, and colored lines on the screen show the type of arm movement to be made. Additional models of both genders also perform the moves in the background. During minigame sections of the game, players can see the avatar they created perform some of the activities. The screenshot below is from the main dance/aerobics portion of the game. The screen shows time elapsed/remaining and points out of the total possible.

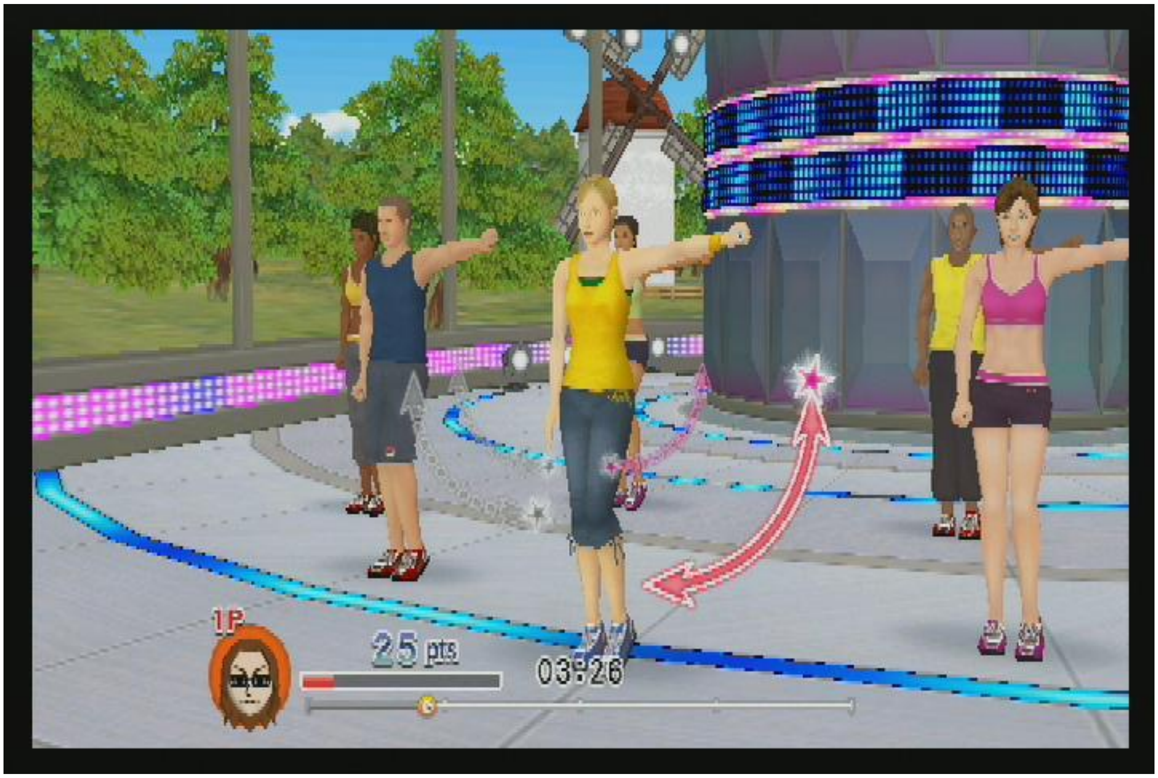

The virtual trainer provides feedback during and after each session. “Rhythm points” are based on accuracy. Combos are streaks of correct movements performed in a row. The game also assigns a ranking of the player’s performance (average, above average, etc.).

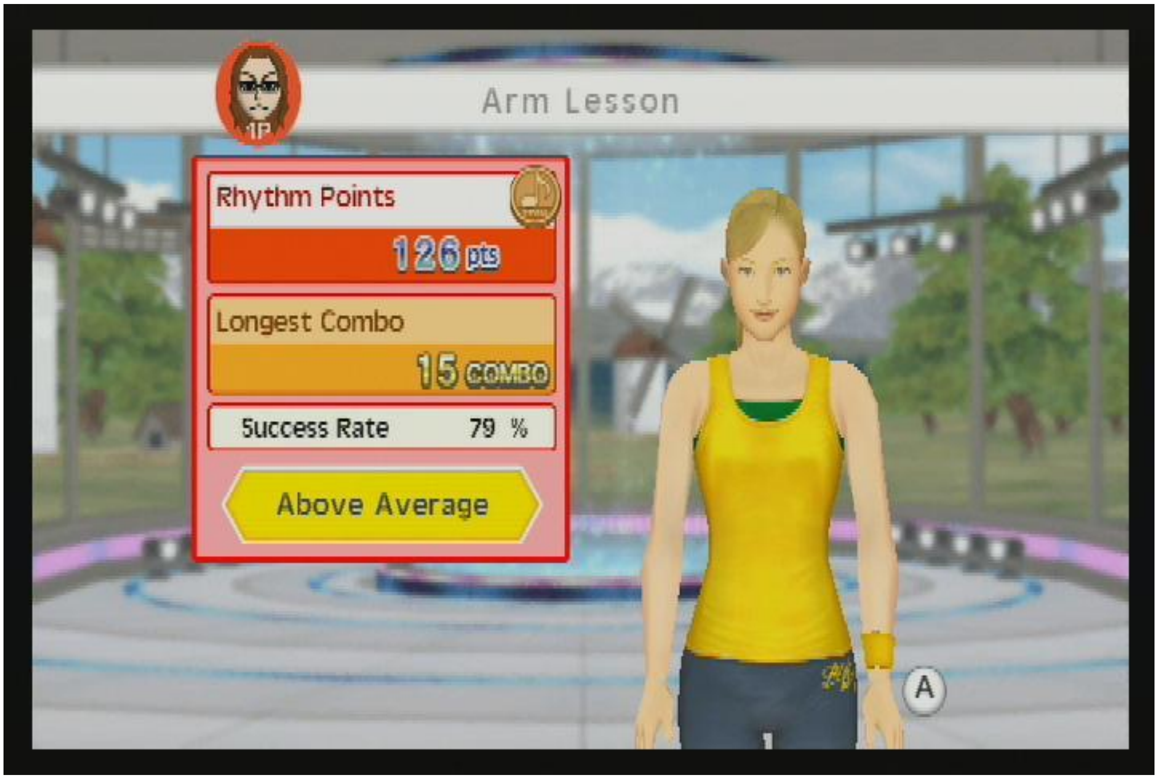

Points are used in a reward structure that assigns a distance walked based upon points and caloric burn. Player avatars walk around the world based upon the distance the game assigns them for each session. Multiple players can compete to finish the game first. The game also includes goals and a calendar. In this game, as in Wii Fit but few other games, players virtually stamp the calendar themselves after completing a session rather than having the game automatically place a stamp.

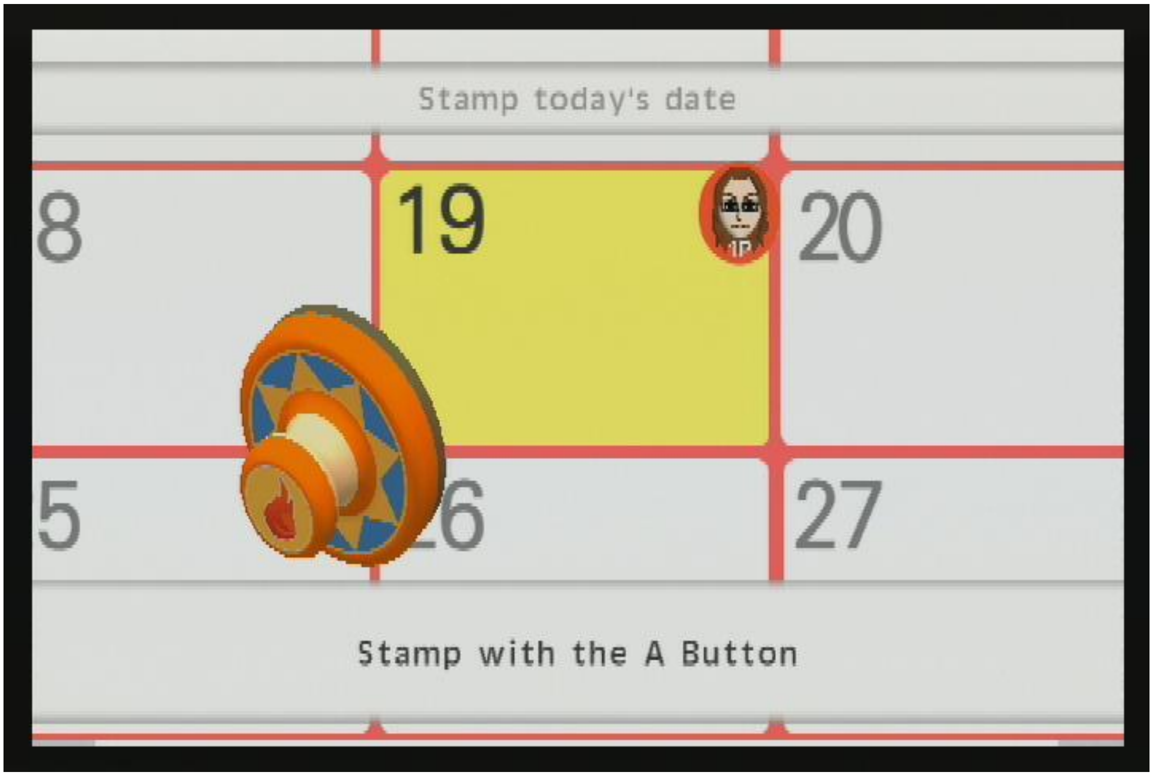

Gold's Gym Cardio Workout

Gold's Gym Cardio Workout (released by Ubisoft in 2009 for the Wii) is a cardio boxing-based fitness game that also includes fitness minigames. Players use a handheld motion sensing controller in each hand. The game uses traditional rhythm gameplay to prompt players to punch. Symbols travel up the screen, and when they reach the area marked with circles, the player is to perform the activity indicated by the symbol. As shown in the screenshot below, the game provides feedback on accuracy (both in terms of each movement, with "perfect," "miss," etc shown on the screen, and in terms of overall score for the session) and progress towards completion of the level. The player's avatar can be seen as a faint outline on the screen. The virtual trainer models the exercises, and the avatar performs them in time with player movements.

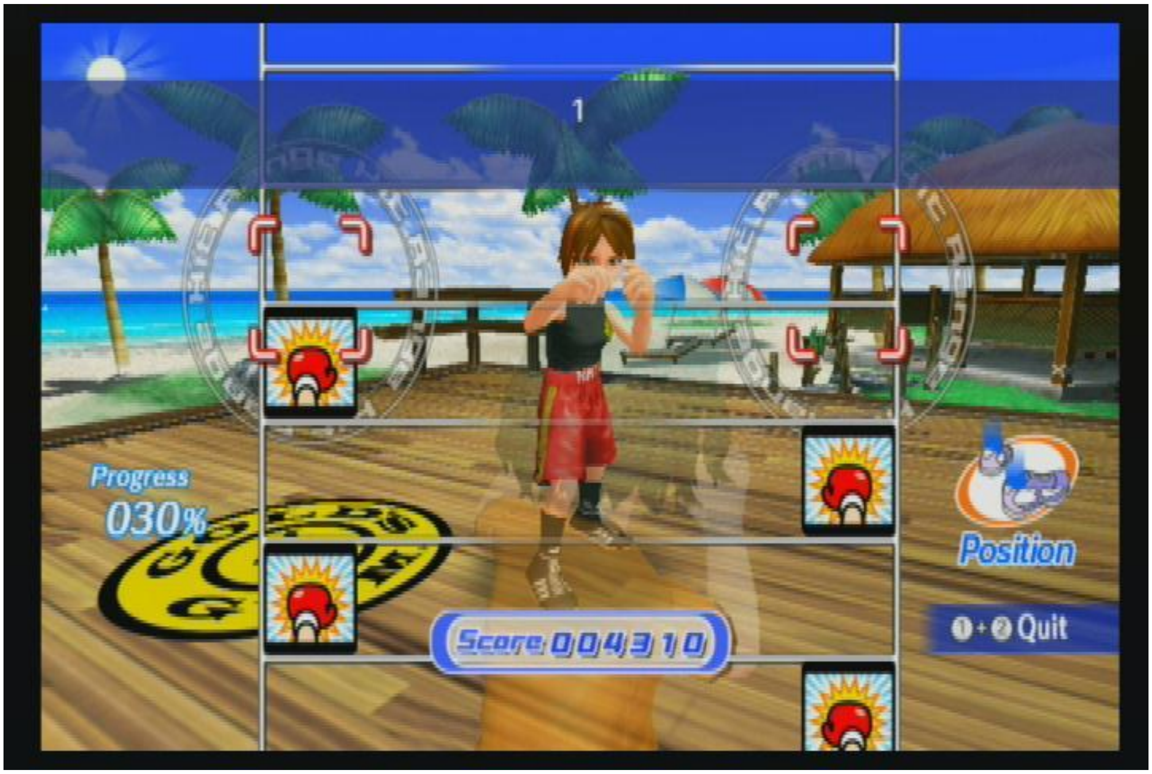

The results screen after completing the session shows time, score, "gold" (which is earned as a reward and can be used to purchase new outfits for the player's avatar), and "physical age." The "physical age" is meant to be general feedback regarding fitness, with a lower age indicating better fitness/performance.

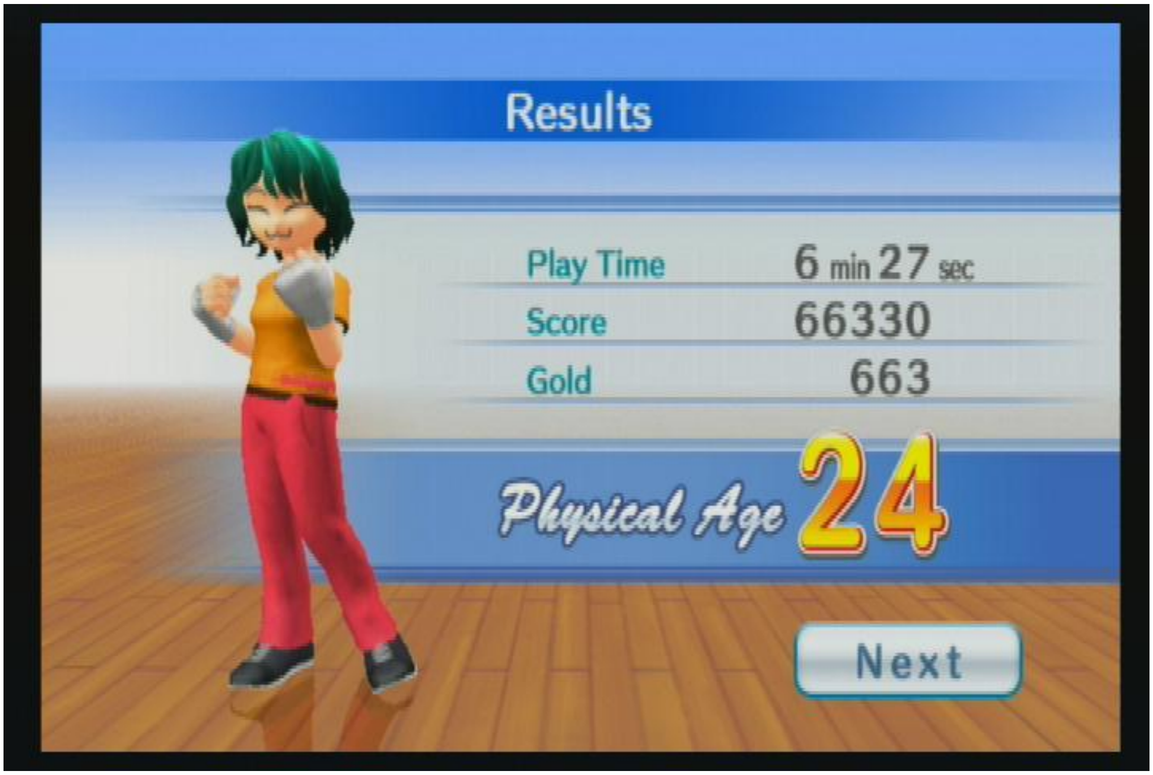

Players can use pre-set programs in the game. This screen shows an intermediate lesson and the information provided about it.

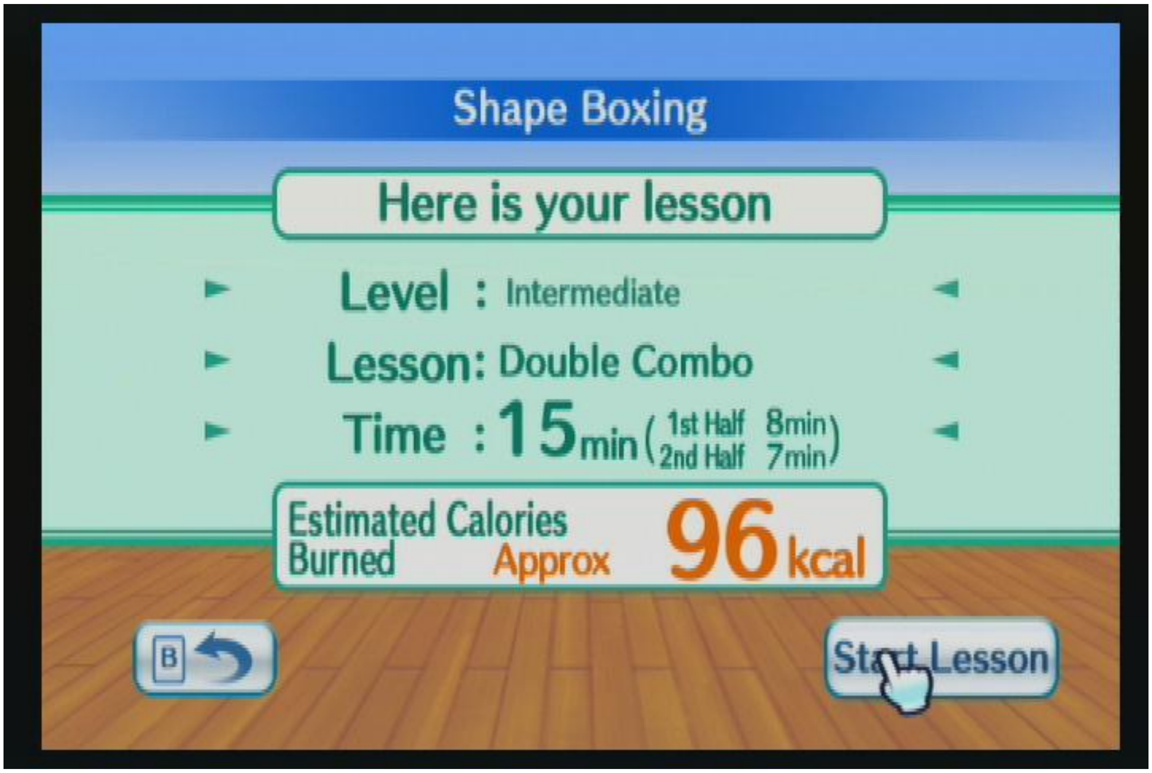

Gold’s Gym Dance Workout

Gold’s Gym Dance Workout, released by Ubisoft in 2010 for the Wii, is relatively dissimilar to the Cardio Workout game released the year before and discussed above. It appears to be more fitness routine focused, with more and a larger variety of behavioral strategies and fewer minigames. The screen below shows one of the introductory screens, which prompts players to set goals in a “personal trainer” section. The menu also shows options for multiplayer workouts, pre-set workout routines, feedback, and rewards.

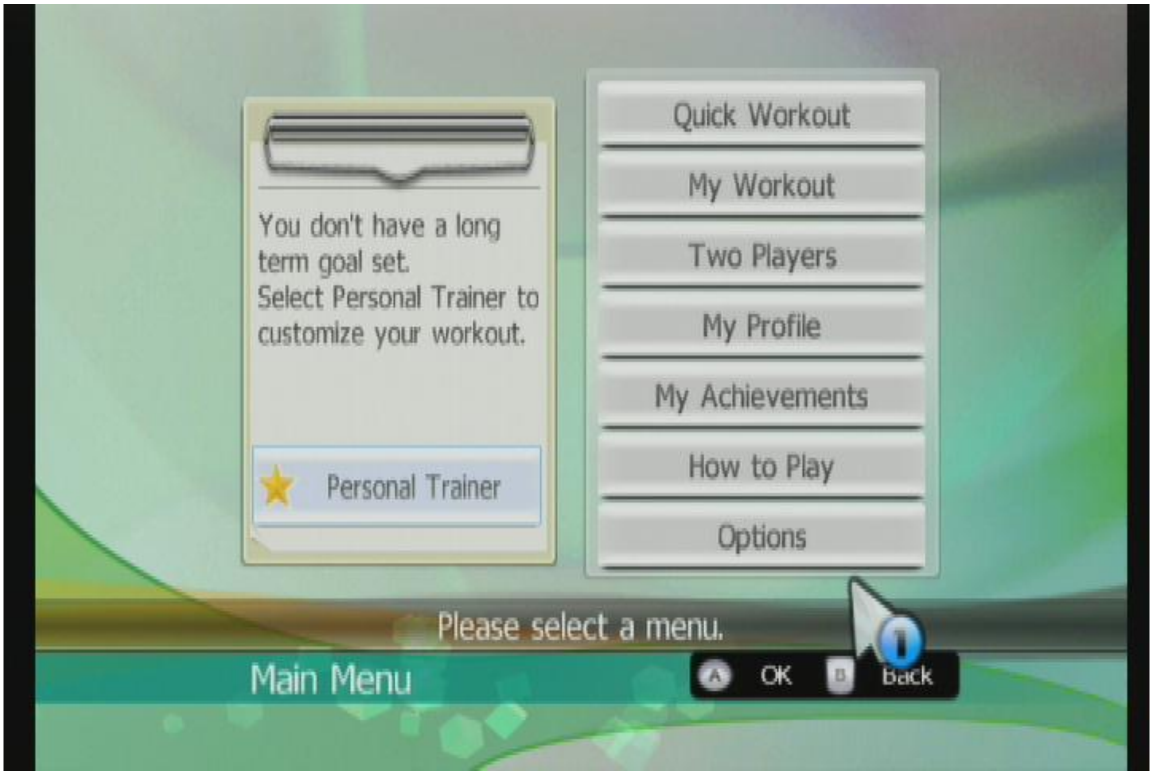

Though there is no true diagnostic pre-test, the game asks questions of players to tailor their experience and recommend goals. Players choose a virtual personal trainer, answer a series of questions, and then are presented with a set of workouts that match their expressed preferences and goals.

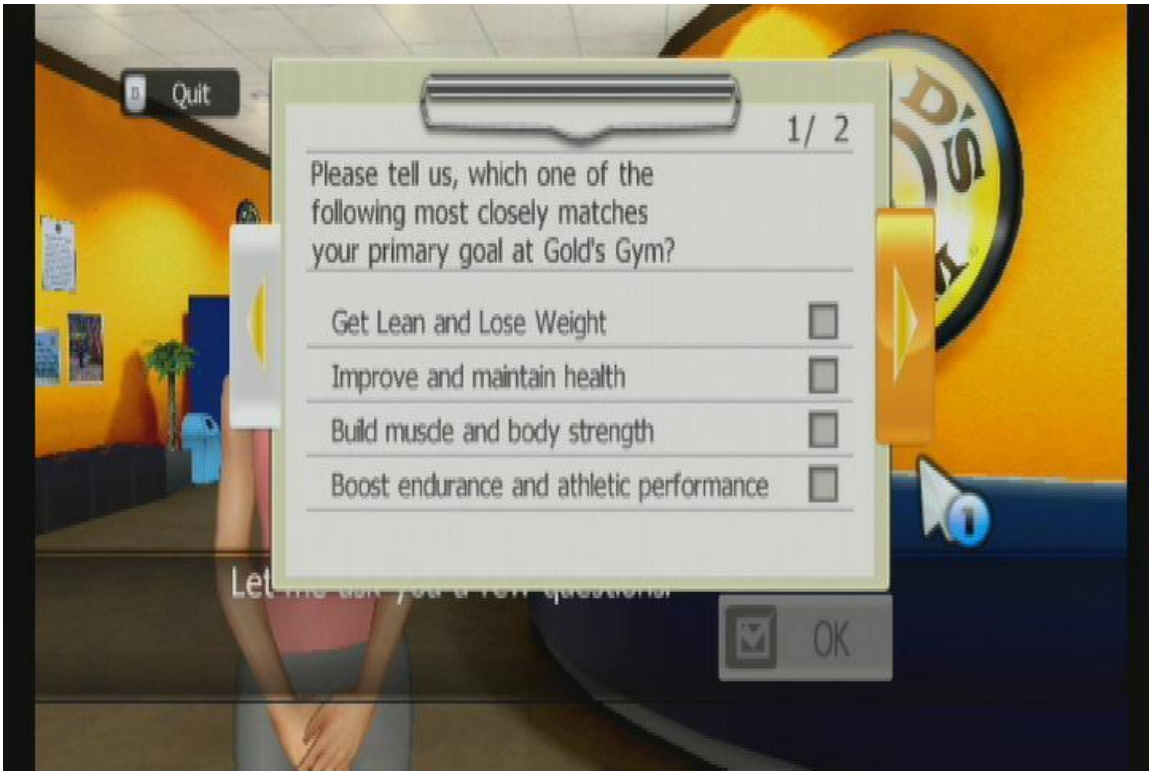

The dance gameplay is similar to that of other dance-based fitness games. The virtual trainer and several other computer graphics-based characters model the movements. Calorie burn is shown at the top left. The four circles at the left light up with words ("miss, "great") to provide accuracy feedback for each set of four movements. A shape to the right shows the player what move is coming up.

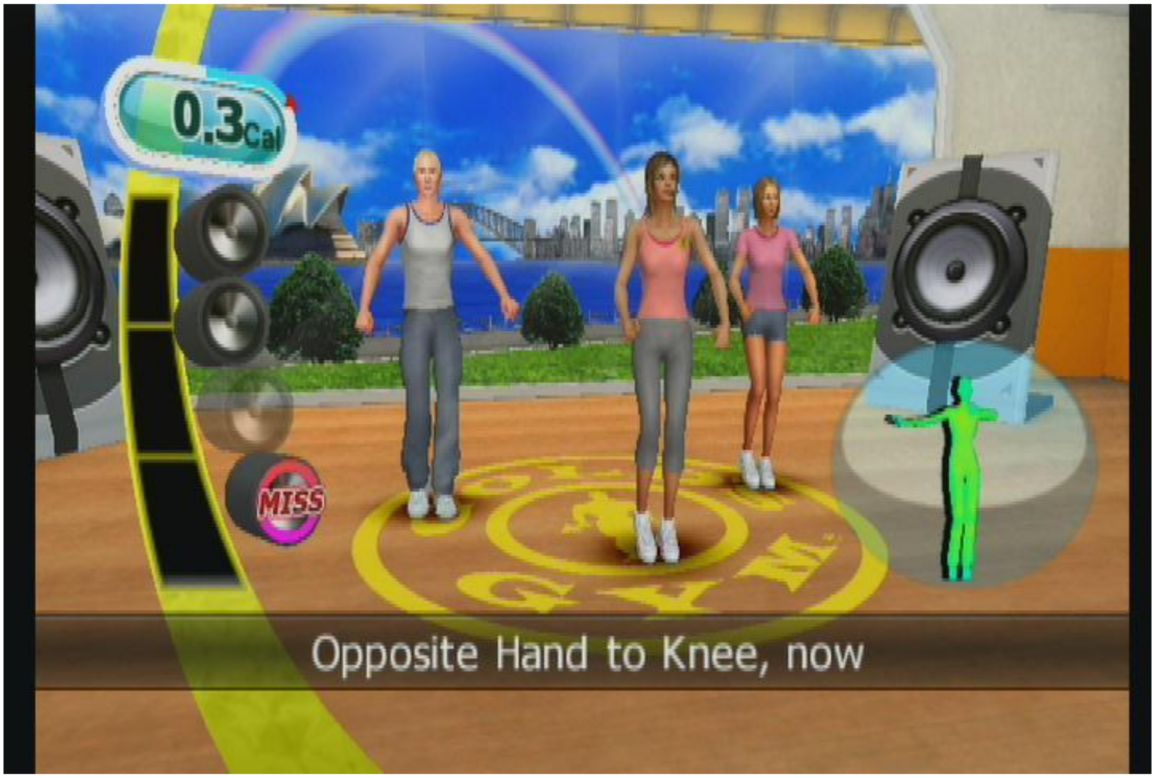

My Fitness Coach

My Fitness Coach, released by Ubisoft in 2008, differs from the other games studied here in that it is much more similar to an interactive workout video than a video game. Rather than using the video game controllers to evaluate movement accuracy, the controllers are used to input basic information and affective responses that are used to tailor a series of aerobic and strength training sessions. The screen below shows part of the extensive diagnostic pre-test. The virtual personal trainer prompts the player to perform several exercise activities, including squats and jumping jacks, for a set period of time. Here, the player is asked to input their resting heart rate and heart rate after performing jumping jacks for two minutes. The results of this initial test are saved and are compared to later identical tests that show changes over time in fitness and strength.

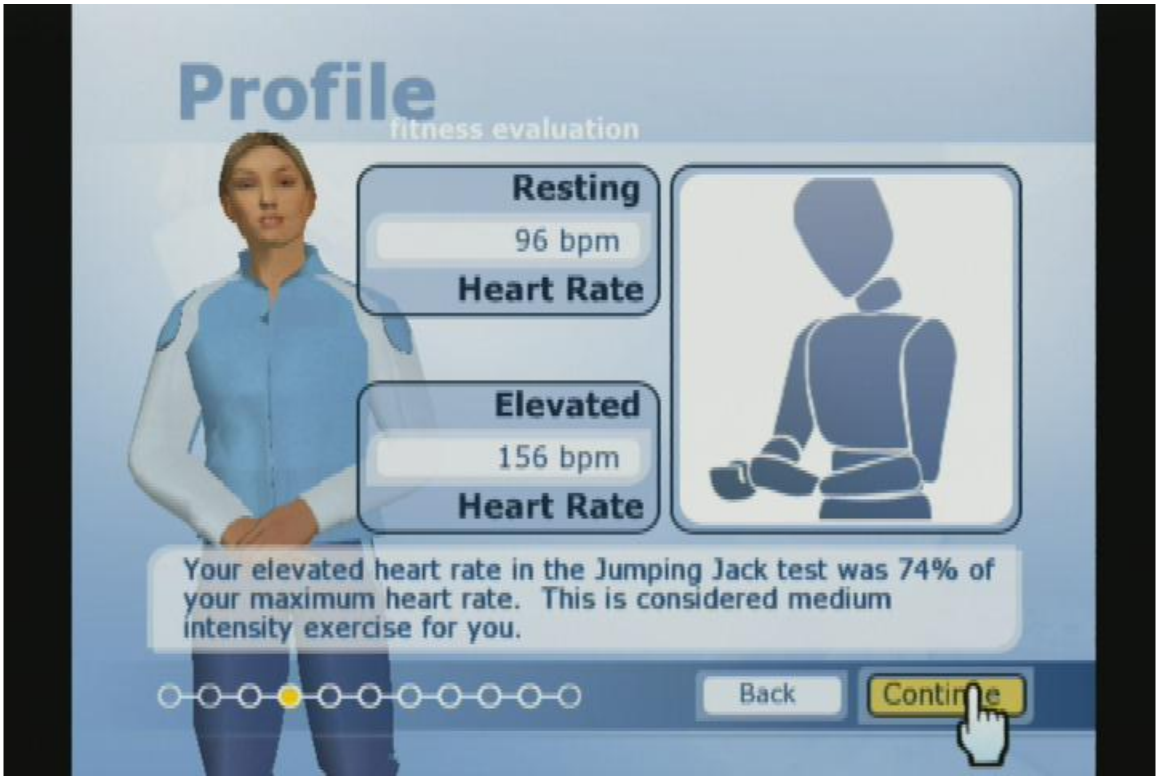

After inputting basic information on weight and performing the diagnostic fitness test, the game “recommends” a particular overall goal and a series of daily goals. The player can reject these goals and choose as they please, however.

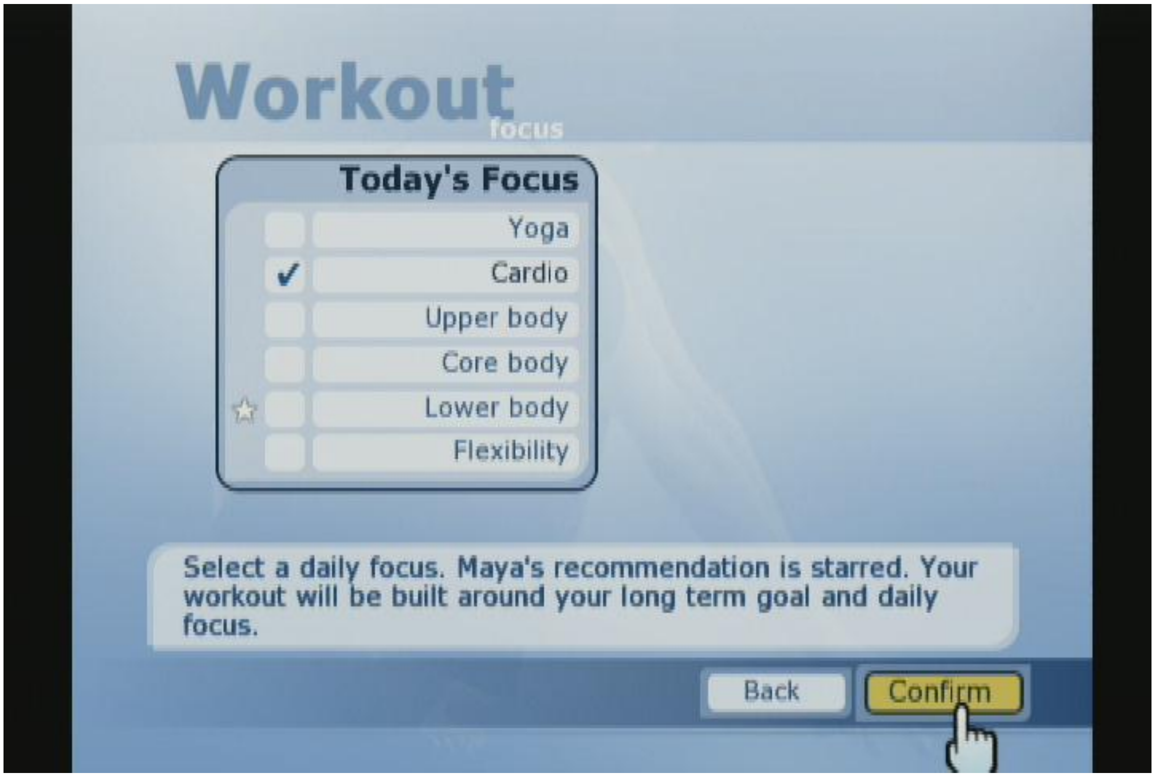

The workout sessions consist of the virtual personal trainer modeling the activities. Players can choose to enter a tutorial, where they can see a move from different angles and for as long as they like. They can also use the controller to indicate whether they would like to decrease or increase the difficulty of the workout.

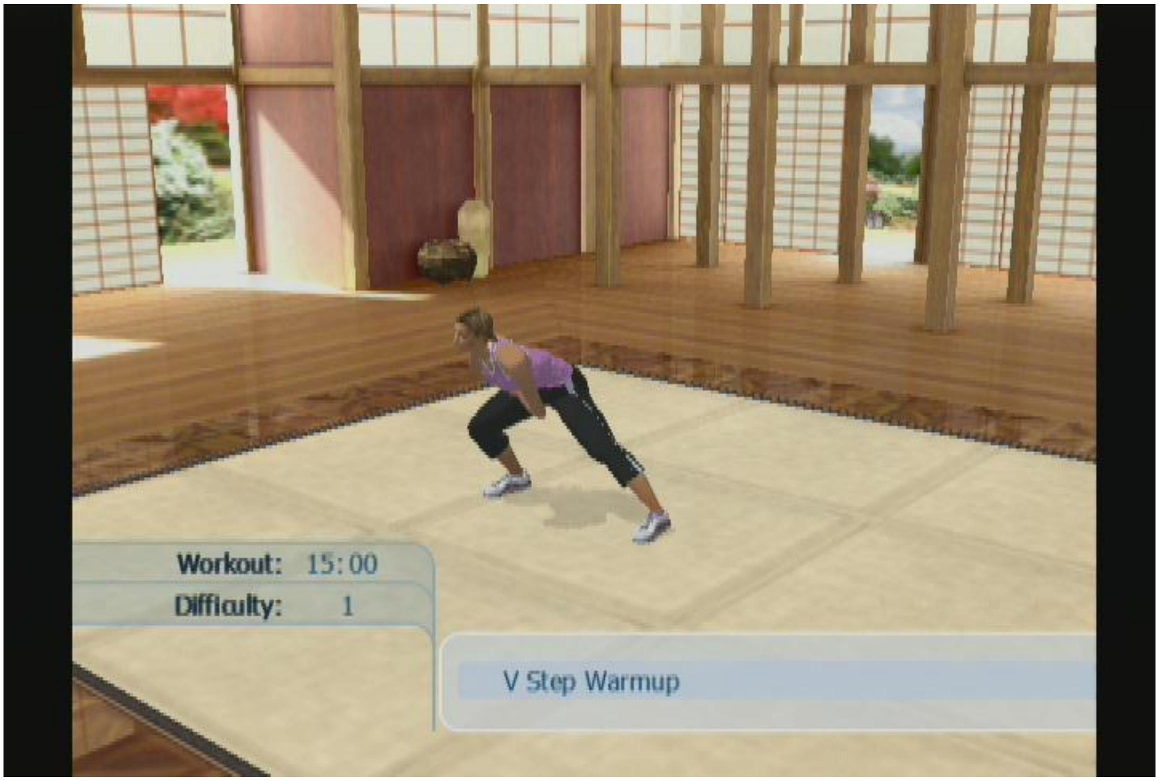

New U Fitness First: Yoga and Pilates

New U Fitness First: Yoga and Pilates was released by Deep Silver in 2010 for the Wii. This game concentrates solely on yoga and pilates sessions. As shown in the screenshot below, players can choose from several different goals at the start of the game. The game provides feedback specific to the goal chosen.

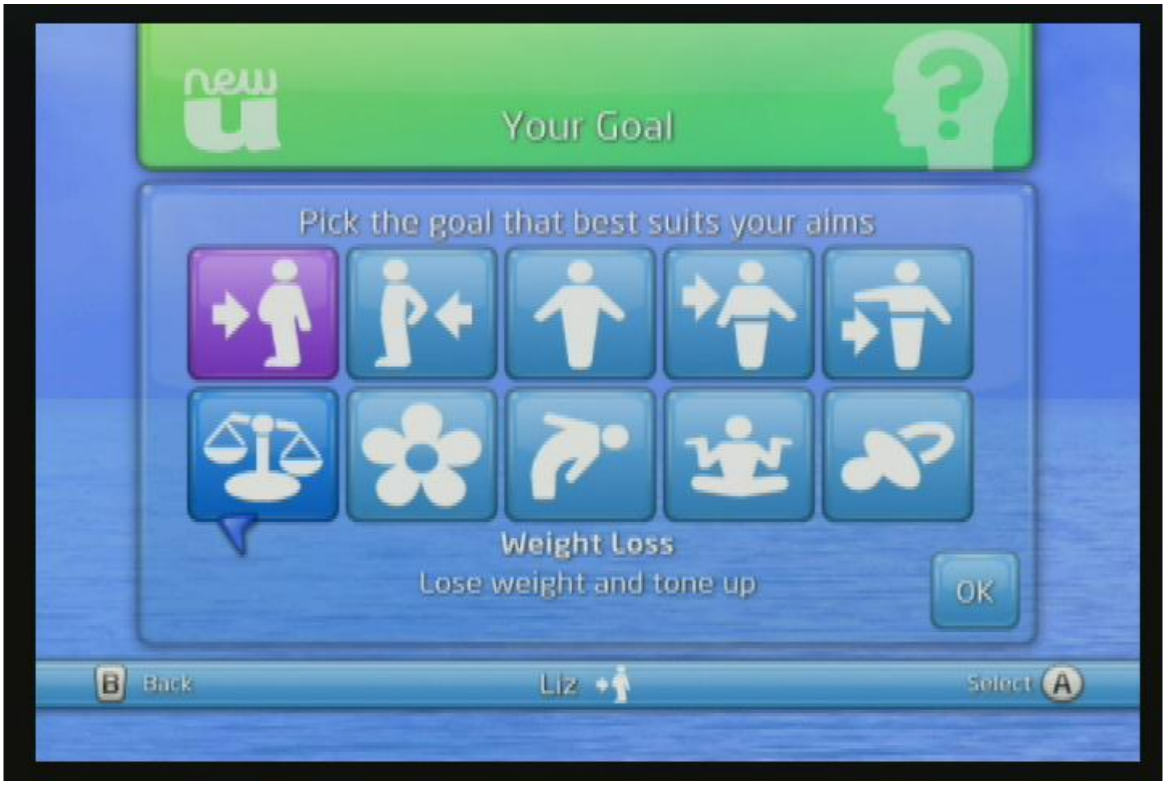

The exercise sessions use video of personal trainers rather then computer graphics. Subtitles are provided in addition to verbal prompts, and the number of repetitions are shown at the top right of the screen. Performance feedback is not provided during the exercises, only once the routines are completed (many of the exercises in the game require players to look away from the screen, which may explain the emphasis on feedback at the conclusion of each set).

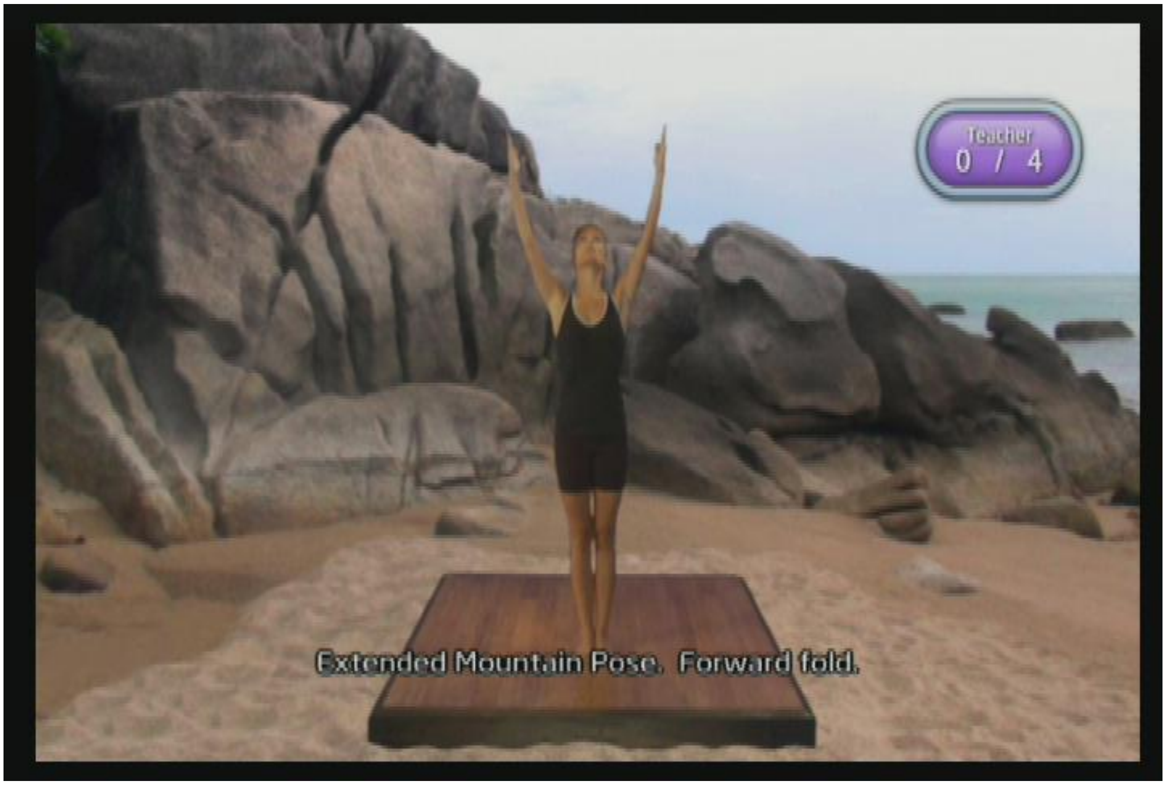

The menu shows the major sections of the game, including toning (yoga and pilates), meditation, "quick class" (i.e., not related to the goal), a place to change profiles to other players, and an options screen.

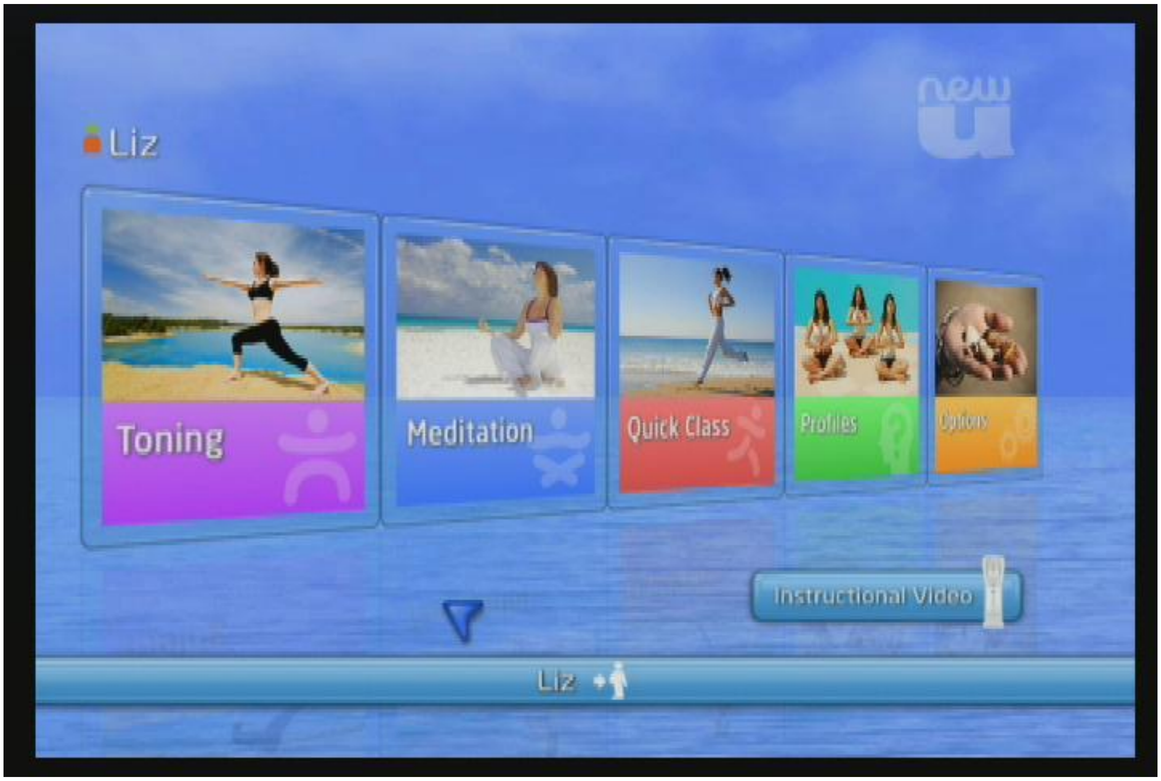

Walk it Out!

Walk it Out! (released by Konami in 2010 for the Wii) is primarily a walking-based fitness game with several minigames included. It is one of the few games studied here that emphasizes a game aspect to the exercises. Players walk in place (walking on a balance board, walking on a dance mat, or placing a handheld motion controller in their pocket) through a virtual town. As they step correctly to the rhythm of the song playing, they earn points that can be redeemed to populate the town with trees, buildings, decorations, and bridges to new areas. The screenshot below shows a player using points to burst a bubble that became a palm tree. The arrows on the screen show options for turning different directions. At the top left are the prompts for stepping. Based on the rhythm of the currently playing song, the blue bars travel across the screen at a certain speed. When they reach the red figure, players are to step. The number indicates the amount of points accumulated. A map of the surrounding area is in the top right corner. A virtual trainer pops up occasionally to provide feedback about distance traveled or to discuss the decoration just earned.

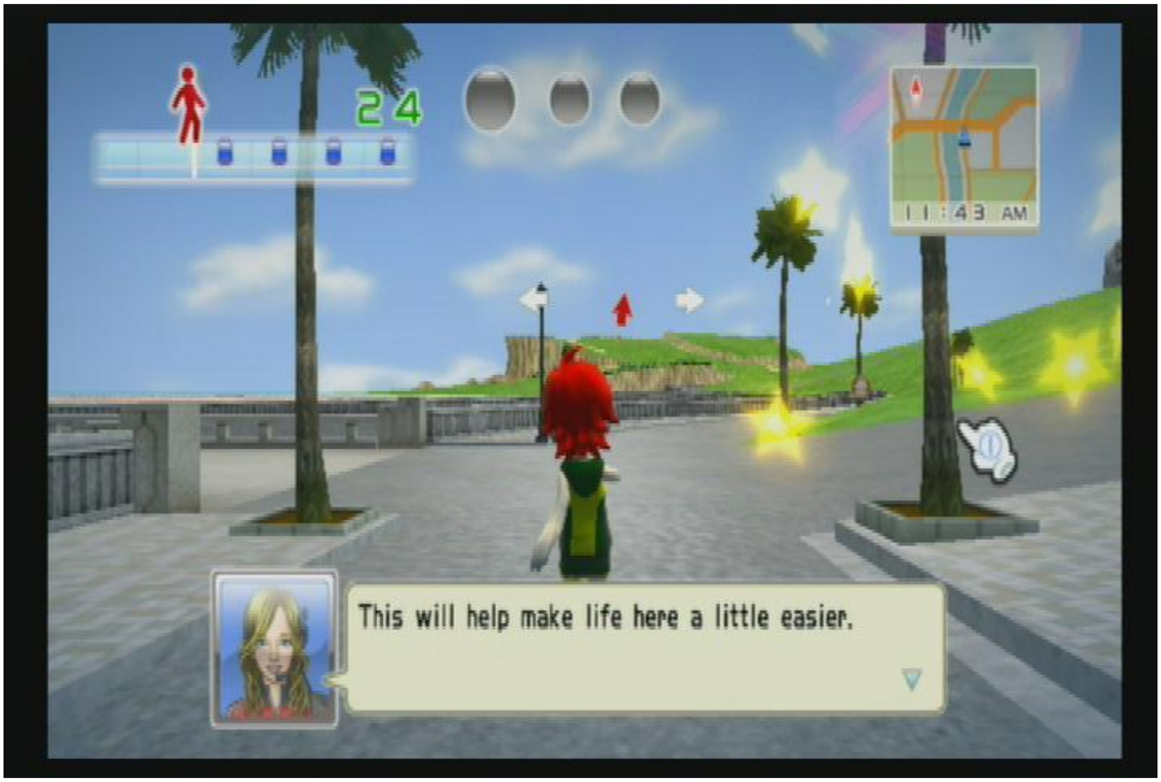

The game provides leaderboards and graphs that allow comparisons over time and across players. Below, the number of calories burned by two players is contrasted.

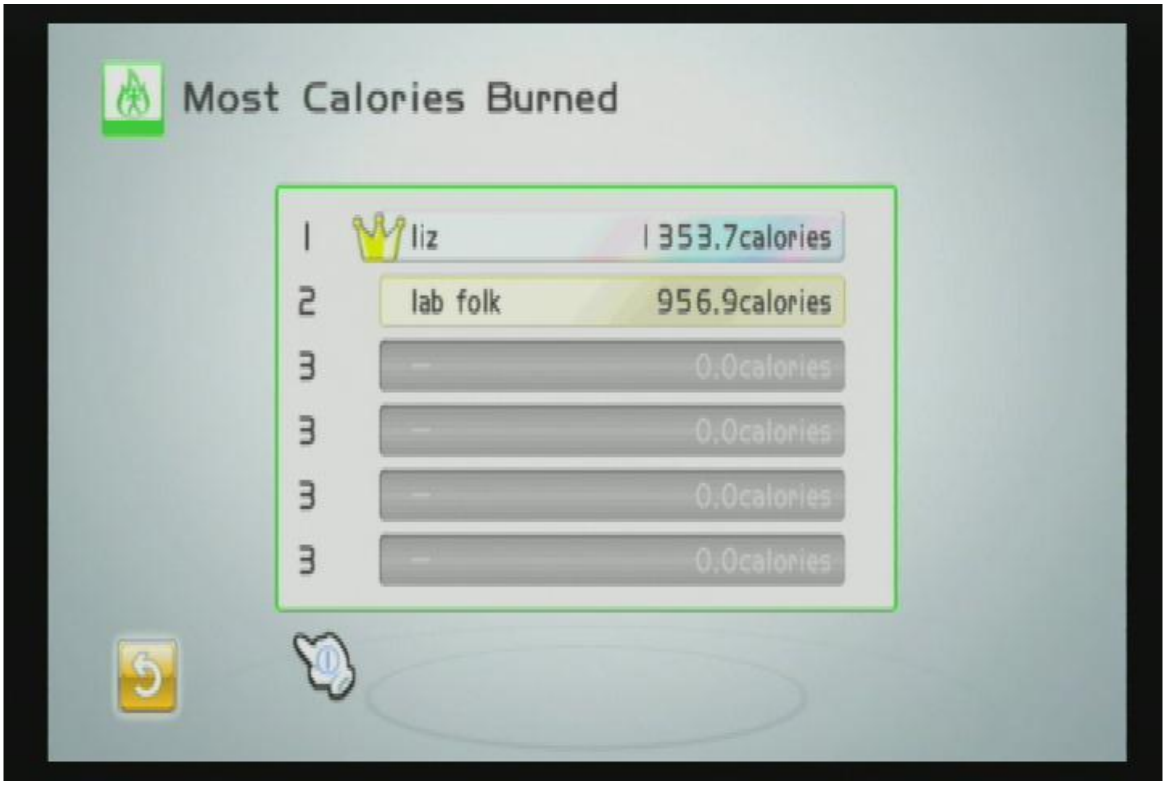

After each session, a feedback screen appears. Feedback is provided in several forms, covering both fitness-related content (steps, calories, distance) and game-related content (portion of the map unlocked, portion of town parts redeemed).

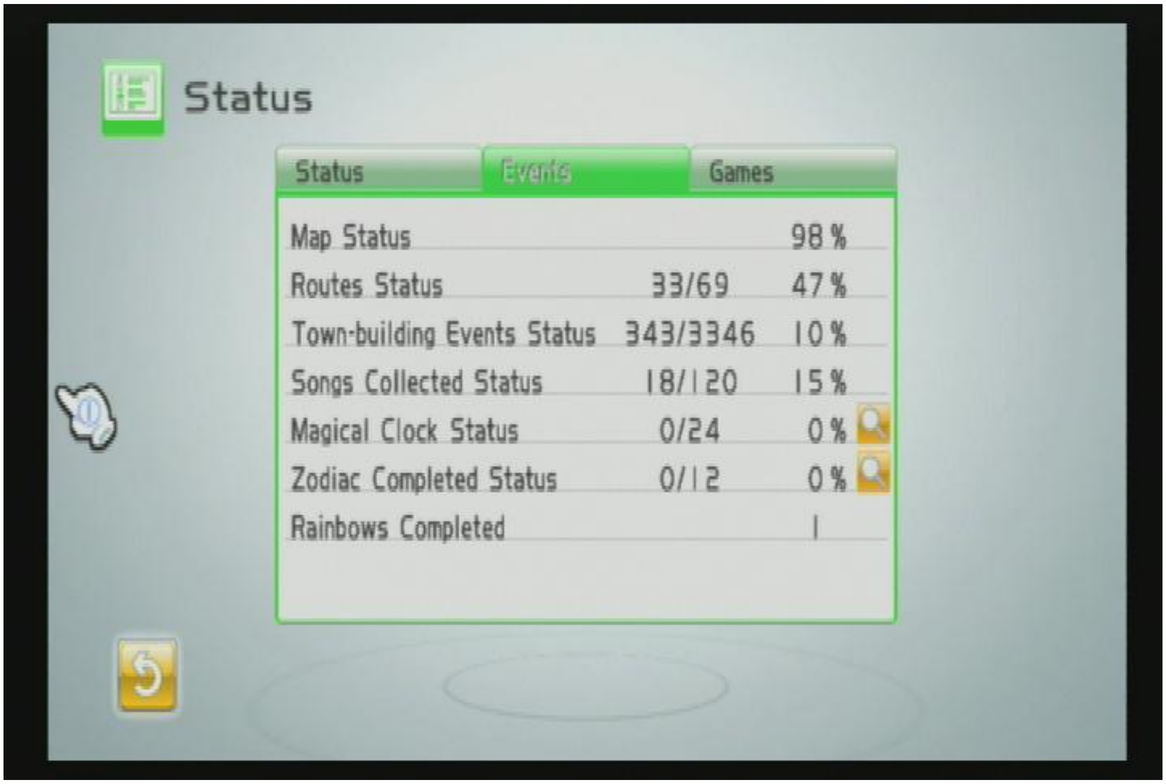

Wii Fit Plus

Wii Fit Plus, released by Nintendo in 2009, is the follow-up to Wii Fit. It includes all of the content from Wii Fit as well as new minigames and behavioral tools. The game is made up of different minigames, some explicitly fitness-oriented (strength, yoga), and some game-based. The screen shot below shows a biking game that requires players to step on the balance board controller and use a handheld motion controller to steer the bike. The bike speed varies based on the player's stepping speed. Their avatar is shown performing the movements, and avatars of other players (including pets) are often shown walking around the town or following the player.

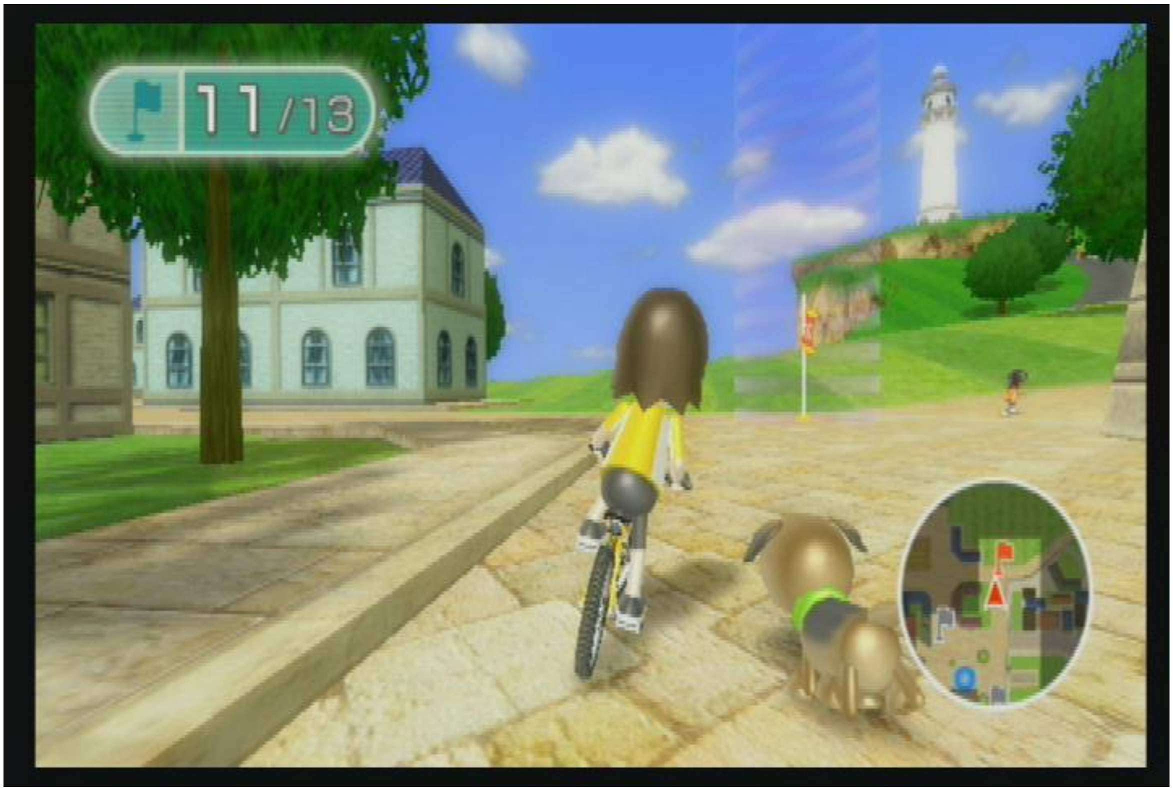

Wii Fit Plus provides graphs of progress for several different variables. The game prompts players to weigh themselves using the balance board controller at every play session and tracks weight over time. It also tracks estimated calories burned playing the game and manually logged "fit credits" from other activities. The screen shot below shows physical activity logged for one day: 30 minutes credited from other activities and 8 minutes spent playing the game. The player avatar at the bottom explains the number of total calories burned from physical activity for the day.

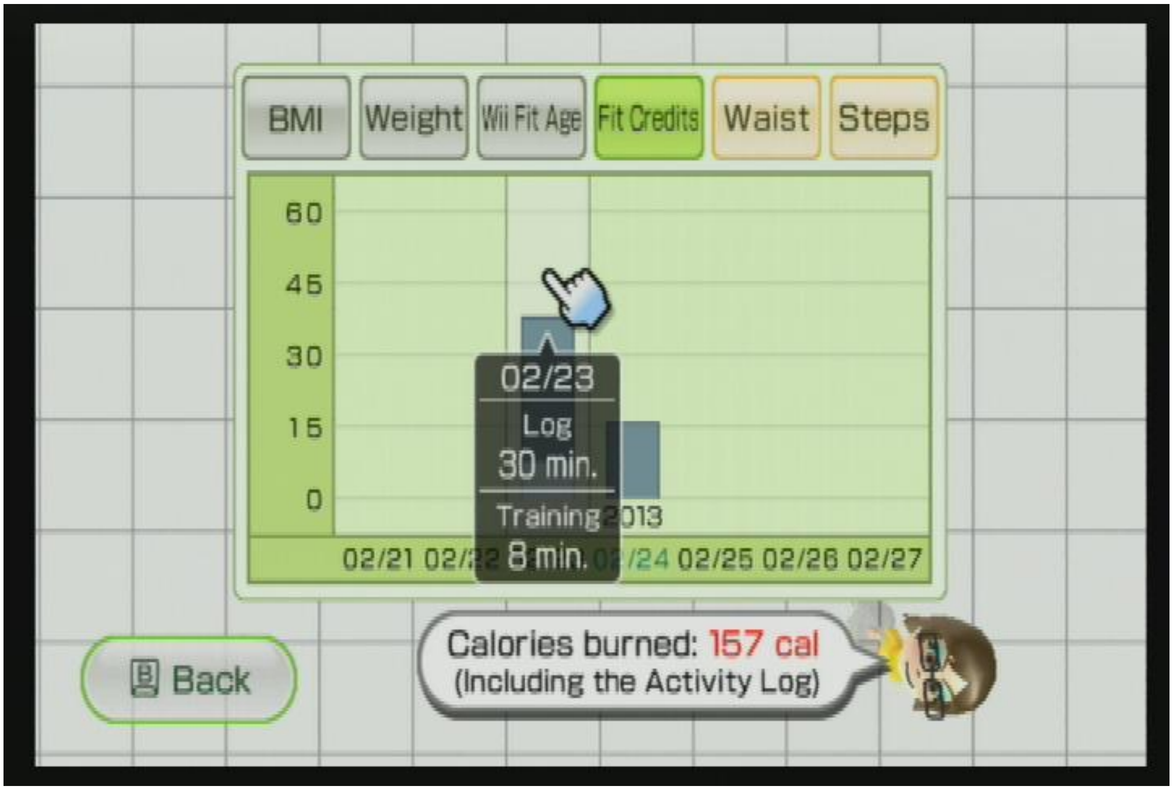

Players can compare their graphs to those of other players. The game also allows tracking of the weight of babies and pets (if held by a registered player so that their weight can be subtracted).

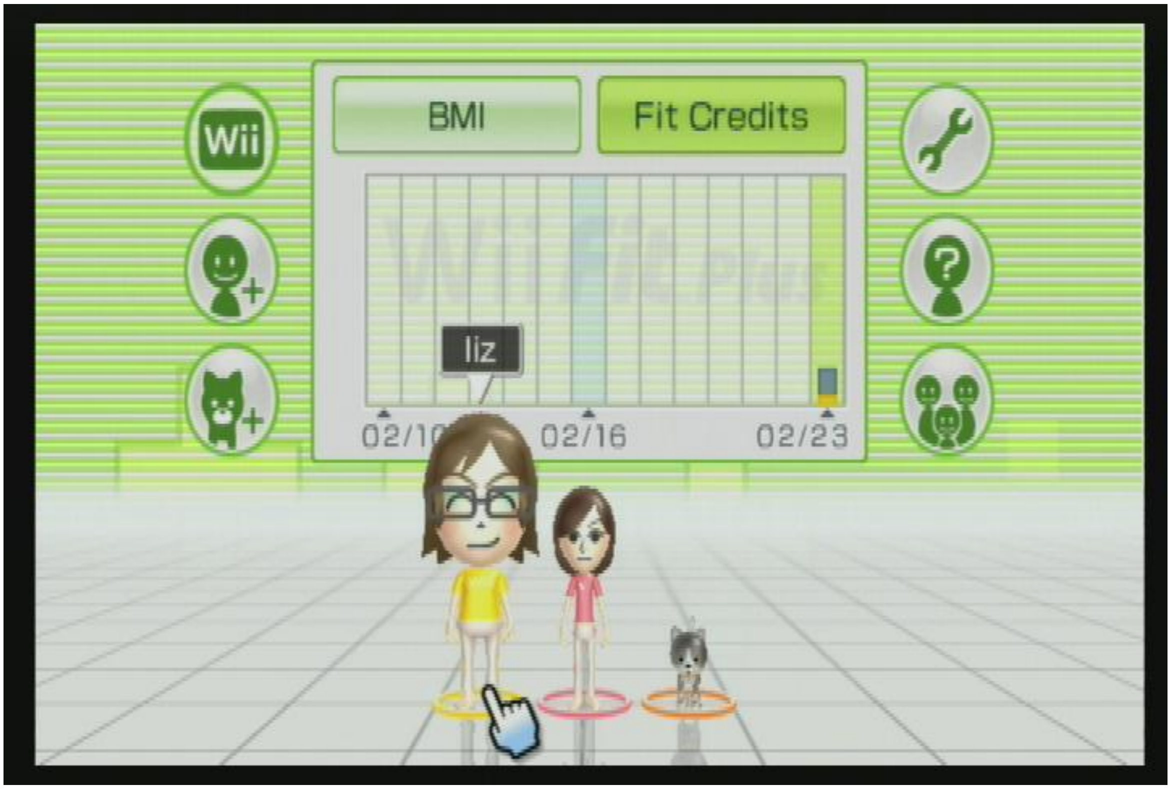

Supplement: Supplementary file 1 [file jmir_v15i5e81_app1.pdf]
