# Supplementary material for: Prevalence of Behavior Changing Strategies in Fitness Video Games: Theory-Based Content Analysis
Source: J Med Internet Res. 2013 May 7;15(5):e81. doi: 10.2196/jmir.2403 (PMC3650924; doi:10.2196/jmir.2403)
Supplement: Supplementary file 2 [file jmir_v15i5e81_app2.pdf]

Multimedia Appendix 2. Behavioral strategies in camera-based games (n = 9).

		Biggest Loser	EA Sports Active 2	Fit in Six	Get Fit With Mel B	Jillian Michaels	The Fight	UFC Trainer	Your Shape	Zumba	Total with strategy
Self-efficacy	Modeling by trainer	■	■	■	■	■	■	■	■	■	9
	Virtual self-modeling	■	■	■	■		■		■		6
	Guided practice		■		■	■	■	■	■	■	7
	Verbal persuasion	■	■		■	■	■	■	■		7
	Accuracy feedback	■	■			■			■	■	5
	Performance feedback	■	■	■	■	■	■	■	■	■	9
	Calorie feedback	■	■		■	■	■	■	■	■	8
	Real-time video	■		■	■	■		■	■	■	7
Self-regulation	Goal-setting	■	■	■	■			■	■		6
	Diagnostic pre-test	■						■	■		3
	Scheduling/planning	■	■					■	■		4
	Workout calendar	■	■	■		■		■	■	■	7
	Comparison to past	■	■	■	■	■	■	■	■	■	9
Other	Social integration	■	■			■	■	■	■	■	7
	Multiplayer	■	■				■	■	■	■	6
	In-game rewards	■		■	■		■	■	■	■	7
	Console rewards	■	■	■	■	■	■	■	■	■	9
	Total tools	16	14	9	11	11	11	15	17	12	
