# Supplementary material for: Prevalence of Behavior Changing Strategies in Fitness Video Games: Theory-Based Content Analysis
Source: J Med Internet Res. 2013 May 7;15(5):e81. doi: 10.2196/jmir.2403 (PMC3650924; doi:10.2196/jmir.2403)
Supplement: Supplementary file 3 [file jmir_v15i5e81_app3.pdf]

Multimedia Appendix 3. Behavioral strategies in controller-based games (n = 9).

		10 Minute Solution	Daisy Fuentes	Exerbeat	Golds Gym Cardio	Golds Gym Dance	My Fitness Coach	New U	Walk it Out	Wii Fit Plus	Total with strategy
Self-efficacy	Modeling by trainer	■	■	■	■	■	■	■		■	8
	Virtual self-modeling	■		■	■	■			■	■	6
	Guided practice	■	■	■	■	■	■	■		■	8
	Verbal persuasion	■					■		■	■	5
	Accuracy feedback		■	■	■	■		■	■	■	7
	Performance feedback	■	■	■	■	■		■	■	■	8
	Calorie feedback			■	■	■	■	■	■	■	7
	Real-time video										0
Self-regulation	Goal-setting			■		■	■	■		■	5
	Diagnostic pretest						■			■	2
	Scheduling/planning	■				■	■				3
	Workout calendar	■		■	■	■	■		■	■	7
	Comparison to past	■	■	■	■	■	■	■	■	■	9
Other	Social integration			■		■	■		■	■	5
	Multiplayer			■			■		■		3
	In-game rewards			■	■	■	■	■	■	■	7
	Console rewards										0
	Total number of tools	8	5	13	9	12	12	8	10	13	
